# A Stress-Activated Transposon in *Arabidopsis* Induces Transgenerational Abscisic Acid Insensitivity

**DOI:** 10.1038/srep23181

**Published:** 2016-03-15

**Authors:** Hidetaka Ito, Jong-Myong Kim, Wataru Matsunaga, Hidetoshi Saze, Akihiro Matsui, Takaho A. Endo, Yoshiko Harukawa, Hiroki Takagi, Hiroki Yaegashi, Yukari Masuta, Seiji Masuda, Junko Ishida, Maho Tanaka, Satoshi Takahashi, Taeko Morosawa, Tetsuro Toyoda, Tetsuji Kakutani, Atsushi Kato, Motoaki Seki

**Affiliations:** 1 Faculty of Science, Hokkaido University, Sapporo 060-0810, Japan; 2 Plant Genomic Network Research Team, RIKEN Center for Sustainable Resource Science, Yokohama 230-0045, Japan; 3 Okinawa Institute of Science and Technology, Onna-son, 904-0495, Japan; 4 RIKEN IMS-RCAI, Yokohama 230-0045, Japan; 5 Iwate Biotechnology Research Center, Kitakami 024-0003, Japan; 6 RIKEN Advanced Center for Computing and Communication, Wako 351-0198, Japan; 7 National Institute of Genetics, Mishima 411-8540, Japan; 8 Kihara Institute for Biological Research, Yokohama City University, Yokohama 244-0813, Japan; 9 Core Research for Evolutional Science and Technology, Japan Science and Technology, Kawaguchi 332-0012, Japan

## Abstract

Transposable elements (TEs), or transposons, play an important role in adaptation. TE insertion can affect host gene function and provides a mechanism for rapid increases in genetic diversity, particularly because many TEs respond to environmental stress. In the current study, we show that the transposition of a heat-activated retrotransposon, *ONSEN*, generated a mutation in an abscisic acid (ABA) responsive gene, resulting in an ABA-insensitive phenotype in *Arabidopsis*, suggesting stress tolerance. Our results provide direct evidence that a transposon activated by environmental stress could alter the genome in a potentially positive manner. Furthermore, the ABA-insensitive phenotype was inherited when the transcription was disrupted by an *ONSEN* insertion, whereas ABA sensitivity was recovered when the effects of *ONSEN* were masked by IBM2. These results suggest that epigenetic mechanisms in host plants typically buffered the effect of a new insertion, but could selectively “turn on” TEs when stressed.

Plants cannot relocate to avoid stressful environmental conditions; therefore, climatic variation is a major environmental stressor and exerts strong selective pressure on plant populations^1^. In response, plants must either possess phenotypic plasticity or generate enough genetic diversity to adapt^2^.

Transposable elements (TEs) are a major source of genetic variation because their insertions create mutations that can affect coding regions or cause genomic rearrangements^3^,^4^,^5^,^6^. Furthermore, environmental stress activates TEs in plants^5^,^7^,^8^,^9^,^10^,^11^,^12^,^13^,^14^,^15^,^16^, potentially triggering the genetic diversity required to evolve adaptations. However, we currently know little about the exact mechanisms behind stress-activated TEs, including whether they are successfully inherited by future generations.

Although TE-generated mutations are important for adaptive evolution, excessive genomic changes can be harmful. Therefore, plants have also evolved processes that can suppress TE activity. A well-studied epigenetic regulation of TEs is RNA-directed DNA methylation (RdDM)^17^,^18^. In *Arabidopsis,* small interfering RNAs (siRNAs) are synthesized from RNA polymerase IV (PolIV)^19^,^20^,^21^ and eventually form an RNA-induced silencing complex that is involved in DNA methyltransferase recruitment, leading to the *de novo* methylation of target TEs^22^,^23^,^24^,^25^.

Previously, we reported that heat stress activates the retrotransposon *ONSEN* in *Arabidopsis*. Moreover, a transgenerational transposition was observed in the progeny of heat-stressed *nrpd1*, a line of mutants that lack functional PolIV (NRPD1 is a subunit of PolIV)^14^. Other experiments in *Arabidopsis* created *ONSEN*-integrated progeny in heat-stressed *nrpd1* mutants^14^,^26^. Because *ONSEN* insertions generally alter gene expression, we hypothesized that *ONSEN*’s gene-targeting transposition could generate novel stress-responsive regulatory genes. In the present study, we induced *ONSEN* transposition in *nrpd1* mutants via heat stress to determine stress tolerance in *ONSEN*-integrated progenies. We used a plant hormone, abscisic acid (ABA), that plays an important role in plant responses to environmental stress^27^. The application of exogenous ABA has been used to mimic osmotic stress. Increased ABA levels induce the expression of many genes that possibly play multifaceted roles in response to osmotic stress^28^. Nearly 10% of the protein coding-genes in *Arabidopsis* are regulated by ABA^29^.

We investigated the mechanism of ABA-insensitive phenotypes in F2 progenies by focusing on the transcriptional regulation of ABA-responsive genes that possessed an *ONSEN* insertion. In *Arabidopsis,* some intronic TEs within transcribed genes are marked by repressive epigenetic modifications, and splicing of the TE-containing intron is promoted by the nuclear protein INCREASE IN BONSAI METHYLATION2 (IBM2)^30^,^31^. IBM2 controls one of the *ONSEN* copies *AT1G11265*, embedded in the intron of F-box protein *AT1G11270* 30. Here, we show that *ONSEN*-integrated progenies in *Arabidopsis* are stress tolerant and the effects of an *ONSEN* insertion are regulated by the host plant.

## Results

### Transgenerational transposition of *ONSEN* resulted in ABA-insensitive plants

**Figure 1:**
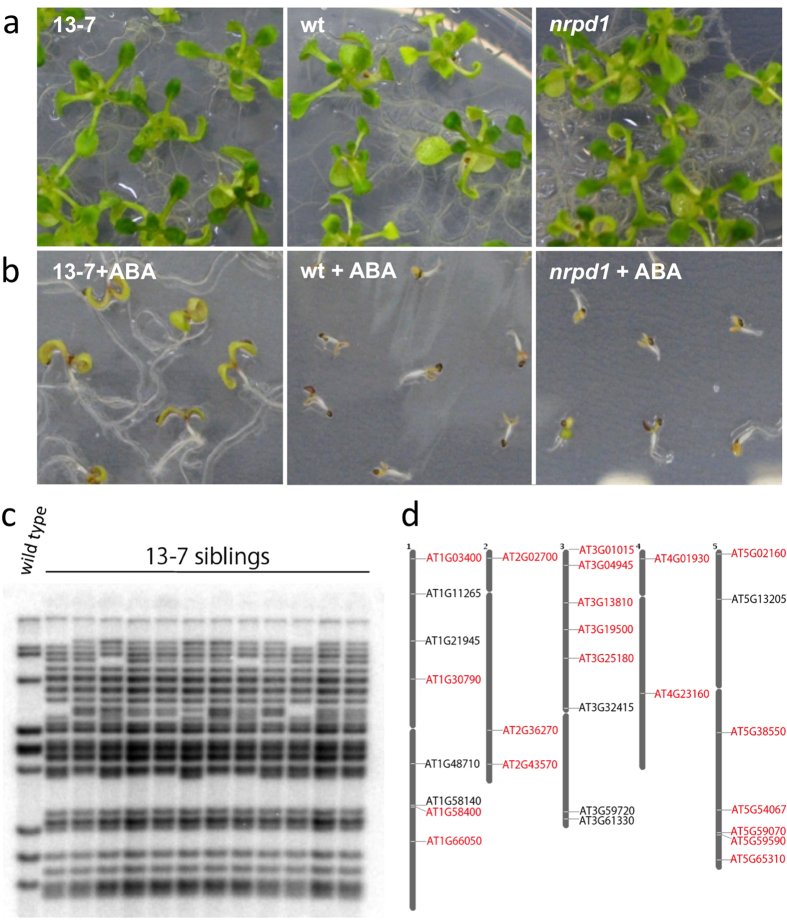
ABA-insensitive progenies with *ONSEN* copies. (**a**,**b**) Phenotypes of young seedlings growing under normal conditions (**a**) and under 2 μM ABA (**b**). 13–7; *ONSEN*-integrated mutant; wt, wild type; *nrpd1*, original *nrpd1* mutant line. (**c**) Southern blot analysis revealed that several new copies of *ONSEN* were detected in ABA-insensitive progenies (13–7). Each lane represents an individual progeny produced from a single heat-stressed *nrpd1*. The leftmost column represents a wild-type progeny growing under normal conditions. (**d**) Mapping of *ONSEN*-integrated genes in a mutant line. Twenty new copies were mapped within genes on the *Arabidopsis* genome in line 13–7. The loci names with red letters indicate the new insertions, while the black letters indicate the original eight *ONSEN* copies.

The *ONSEN*-integrated progeny in heat-stressed *nrpd1* mutants were seeded on an MS medium containing ABA. ABA is closely associated with cellular dehydration processes in seed maturation and vegetative growth. We also tested heat, salt, and cold stress. The phenotype we analyzed with ABA was the clearest result of environmental stress responses. The *ONSEN*-integrated mutants that exhibited ABA-insensitive phenotypes were screened 10 d after germination. Out of 8,000 seeds, we found two ABA-insensitive mutants. Mutant phenotypes do not differ from the wild type under normal conditions (Fig. 1a). However, in the presence of ABA the mutants exhibited elevated ABA-resistance (Fig. 1b). A Southern blot analysis of the ABA-insensitive progenies revealed that *ONSEN* copies were inserted into multiple loci (Fig. 1c), suggesting that a new *ONSEN* insertion could affect the expression of an ABA-responsive gene.

### *ONSEN* insertions are associated with euchromatic genes

To identify the *ONSEN* insertion sites, we analyzed the whole genome sequences of the two stress-insensitive mutants using a next generation sequencer. In one mutant (13–7), new *ONSEN* insertions were identified in 24 loci of the sequenced genome (Fig. 1d, see Supplementary Table S1): 20 were located within genes and 14 were inserted within exons. In the other mutant (19–4), the new copies were inserted into 21 loci: 13 were located within genes and eight were inserted within exons (see Supplementary Fig. S1, Supplementary Table S2). We then assessed the *ONSEN*-targeted genes based on all annotated genes in the TAIR10 database (see Supplementary Table S3) to understand *ONSEN* target site preferences. The average size, exon number, and exon size of the targeted genes did not differ from the reference genes. However, the average intron size of the targeted genes was significantly smaller than that of the reference genes. These results indicate that a new *ONSEN* copy can target genes independent of DNA sequence. Instead, the transposon may be directed by gene structure on euchromatic regions.

### *ONSEN* target sites

A heat-activated gene is a possible candidate for an *ONSEN* target site because transgenerational transpositions occurred in heat-stressed plants. To test this hypothesis, we compared heat-stressed versus normal expression levels of *ONSEN*-targeted genes in both wild type and *nrpd2* mutants (see Supplementary Table S4). We chose *nrpd2* mutants because plants deficient in NRPD2 (a subunit of PolIV) are hypersensitive to heat exposure^32^. Published microarray data of genome-wide gene expression in heat-stressed wild type and *nrpd2* plants were used for the analysis. In *nrpd2*, *ONSEN*-targeted gene expression levels were not upregulated by heat stress; rather, 6 out of 33 target genes were down-regulated (see Supplementary Table S4). Although heat activation of *ONSEN*-targeted genes in *nrpd1* was not analyzed, these results indicate that *ONSEN* transposition does not require heat activation of the target site.

We previously reported that *ONSEN* insertion must take place in flowers^33^. To determine whether the insertions identified in the current study correspond to genes that are activated during flower development, we analyzed genes that are targeted by *ONSEN* using microarray data (see Supplementary Table S5). The results showed that the genes were not transcribed at a specific stage during flower development and did not correspond to the time when transposition events previously resulted in stable inheritance.

### Transposition of heat-activated *ONSEN* into ABA-responsive genes

To identify the gene(s) responsible for the ABA-insensitive phenotype, we focused on *ONSEN* insertions in ABA-responsive genes. Whole genome sequencing data of the 13–7 line revealed an *ONSEN* insertion in the first intron of *ABSCISIC ACID-INSENSITIVE5* (*ABI5*) in the 13–7 line: located on chromosome 2 in position 15,207,390 on an AGI (*Arabidopsis* Genome Initiative) map, *ABI5* encodes a basic leucine zipper transcription factor involved in ABA signaling during seed maturation and germination^34^,^35^,^36^,^37^ (see Supplementary Fig. S2a). In the 19–4 line, an *ONSEN* insertion was detected on an exon of *ABSCISIC ACID-INSENSITIVE4* (*ABI4*). Located on chromosome 2 in position 16,797,945 of an AGI map, *ABI4* is a member of the APETALA2 (AP2) domain family of transcriptional regulators. Mutant *abi4* alleles have been identified in a variety of screens including ABA-resistant germination and salt-resistant germination^38^,^39^,^40^ (see Supplementary Fig. S2b).

To confirm an *ONSEN* insertion in the *ABI5*- and *ABI4*-coding regions, PCR was conducted to amplify the target region. The results indicate that a full-length *ONSEN* was integrated into both *ABI5* and *ABI4* (see Supplementary Fig. S2c,d).

The *ONSEN* family in the *A. thaliana* ecotype *Columbia* consists of eight full-length copies distributed over chromosomes 1, 3, and 5. To determine which member of the family was transposed into *ABI5* in 13–7 and *ABI4* in 19–4, a 700-bp sequence of the long terminal repeat (LTR) and the coding region of the inserted *ONSEN* were scored for element-specific single nucleotide polymorphisms that distinguish the eight genomic templates. We found that the *ONSEN* insertion sequence in 13–7 and 19–4 was assigned to *AT1G11265* and *AT3G61330*, respectively (see Supplementary Fig. S3). These two copies contained intact LTRs and are capable of forming extrachromosomal copies that can potentially integrate into new genomic sites^41^.

### An *ONSEN* insertion disrupted ABA-responsive genes in the mutant progenies

**Figure 2:**
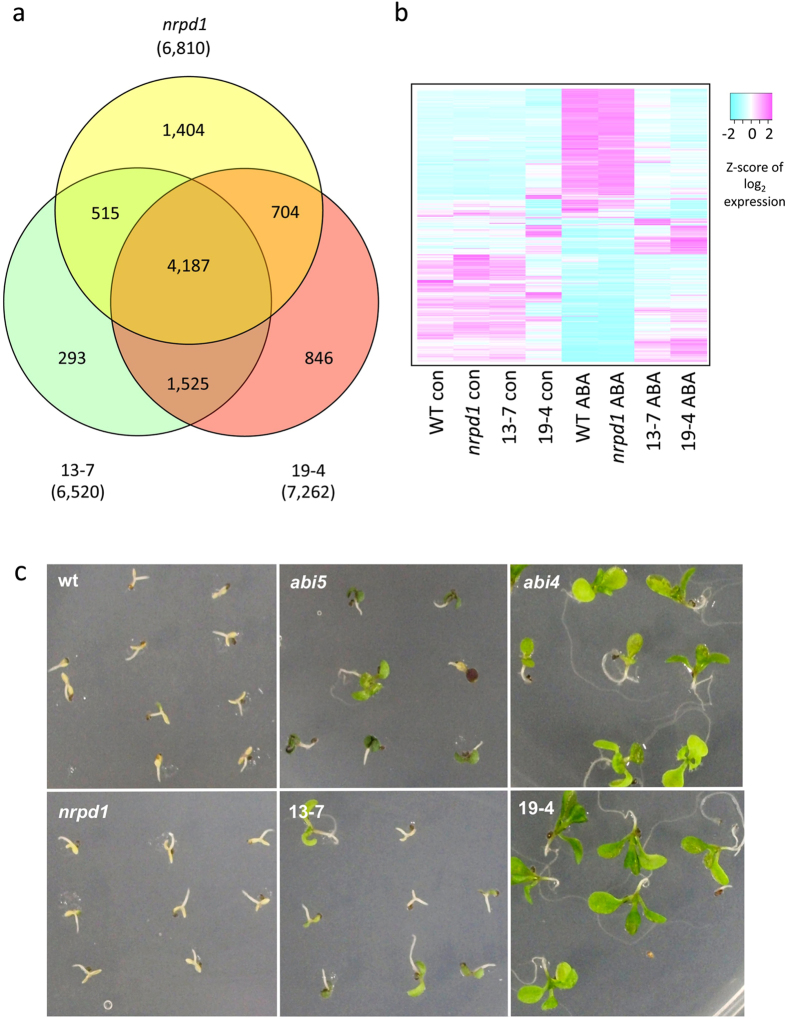
Genome-wide gene expression analysis and ABA insensitive phenotype of the *ONSEN*-integrated lines. (**a**) Venn diagram of differentially expressed genes in 13–7, 19–4, and *nrpd1* under ABA stress. Microarray analysis identified significant differentially expressed (expression change was 1.5-fold or 0.67-fold, FDR <0.05) genes of 13–7 and 19–4 compared with parental *nrpd1* under ABA treatment (green and red). ABA-responsive genes were identified by comparing ABA treated-*nrpd1* with non-treated-*nrpd1* (yellow, expression change was 1.5-fold or 2/3-fold, FDR <0.05). (**b**) Expression heat map of significant differentially expressed genes in 13–7 and 19–4. Genes that exhibited significant differential expression in (**a**) were used. The heat map shows the expression levels in wild type, *nrpd1*, 13–7, and 19–4, with or without ABA treatment. (**c**) ABA-insensitive phenotypes at germination. The phenotype of *abi5* and *abi4* were similar to 13–7 and 19–4 respectively.

**Figure 3:**
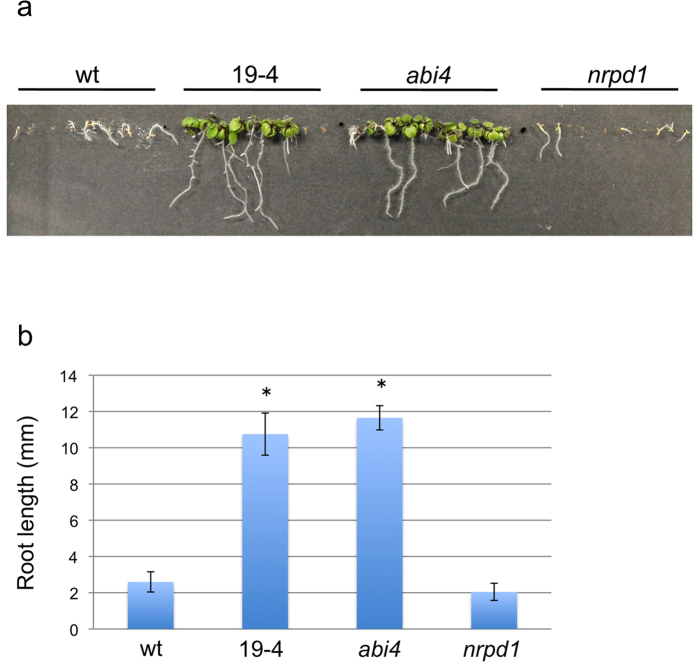
Salt-tolerance phenotype of 19–4. (**a**) Two-week old seedlings of 19–4 and *abi4* mutant showed root elongation under 100mM NaCl. (**b**) Comparison of root length of two-week old seedlings growing under salt stress (n = 10). Statistic analysis showed the root length of 19–4 and *abi4* mutant was significantly longer than that of wild type. Asterisks mark significant difference (P < 0.05).

To understand how *ONSEN* insertions affect genes, genome-wide gene expression analyses were performed on the *ONSEN-*integrated lines using a microarray. The microarray analyses identified 6,520 and 7,262 genes that were expressed differentially under ABA stress in 13–7 and 19–4, respectively (expression change was at least 1.5-fold for upregulated genes and 0.67-fold or lower for down-regulated genes, FDR <0.05, Fig. 2a). We also found that 4,702 genes (72%) in 13–7 and 4,891 genes (67%) in 19–4 overlapped with the ABA-responsive genes in *nrpd1* (Fig. 2a). Most of the differentially expressed genes in the two mutant lines exhibited low ABA-sensitivity (Fig. 2b), suggesting that both 13–7 and 19–4 were critically deficient in processes involving ABA response. ABI4 and ABI5 are both transcription factors that regulate seed-specific ABA-inducible genes during germination^37^,^39^,^42^. 13–7 and 19–4 exhibited ABA-insensitive phenotypes at germination that were consistent with the phenotype in *abi5* and *abi4* mutants (Fig. 2c). In addition, 72 out of 95 genes and 28 out of 59 genes that were directly regulated by ABI4 and ABI5 were suppressed in 19–4 and 13–7, respectively (see Supplementary Fig. S4). The probability frequency of ABI5/ABI4 targets or differential ABA-response genes in 13–7 and 19–4 was examined by a two-tailed binomial test. The statistical test indicated that the representation of the overlap between ABI5/ABI4 targets and the differential ABA-response genes in the 13–7 and 19–4 lines was significantly high (p-value: 2.2 × 10^−1616^ and 1.4 × 10^−1747^ respectively). Previous research investigating the suppression of *ABI4* and *ABI5* expression has shown similar results in downstream genes^43^. A mutation in *ABI4* was shown to increase salt tolerance^44^,^45^,^46^. To determine the salt tolerance in an *ONSEN* insertion mutant, we applied salt stress to the seedlings of 19–4. The tested mutant plants led to elongate root compared to wild type when the seedlings were growing on top of agar-solidified medium containing 100 mM NaCl (Fig. 3). In summary, *ONSEN* insertion in these lines created mutant alleles of the affected genes.

### The intragenic insertion of *ONSEN* was cloaked by IBM2

The ABA insensitive phenotype was inherited in the self-fertilizing progeny of 13–7 (see Supplementary Fig. S5), but not when 13–7 was crossed with the wild type (F2 population; see Supplementary Fig. S6b). To test potential trans-effects, 13–7 was backcrossed with *nrpd1*. Of 112 seedlings, 8 showed an ABA-insensitive phenotype and the ABA-insensitive segregant contained the *ONSEN* insertion within *ABI5*; however 20 out of 100 of the *ONSEN* insertion was detected in ABI5 in the F2 (see Supplementary Fig. S7). In contrast, about 25% (24 out of 100) of the F2 population from a cross between 19–4 and the wild type was ABA-insensitive (see Supplementary Fig. S6d).

**Figure 4:**
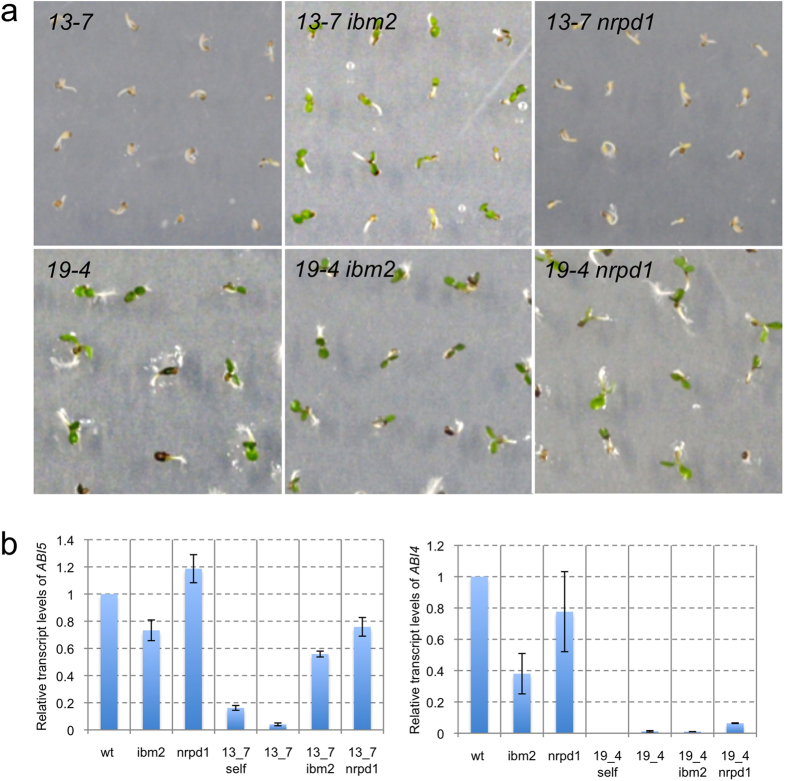
ABA-insensitive phenotype and a transcription level of *ABI4* and *ABI5* in the F3 progeny. (**a**) Phenotype of F3 progenies derived from progenies of a cross between an ABA-insensitive mutant (13–7 or 19–4) and an *IBM2* mutant (*ibm2*) growing under 2 μM ABA. *13–7*, *19–4*: F3 progenies that contain an *ONSEN* insertion in *ABI5* and *ABI4*, respectively. *13–7 ibm2*, *19–4 ibm2*: F3 progenies that contain a mutation in *ibm2* and an *ONSEN* insertion in *ABI5* or *ABI4*, respectively. *13–7 nrpd1*, *19–4 nrpd1*: F3 progenies that contain a mutation in *nrpd1* and an *ONSEN* insertion in *ABI5* or *ABI4*, respectively. (**b**) Transcript levels of *ABI5* and *ABI4* in the F3 progenies. Transcript levels of *ABI5* and *ABI4* in F3 progenies (derived from the progeny of a cross between 13–7 or 19–4 and an *ibm2*) growing under 2 μM ABA.

*ONSEN* was inserted into the first intron of *ABI5* in 13–7 and into the single exon of *ABI4* in 19–4 (see Supplementary Fig. S2a,b). To determine epigenetic regulation of intragenic *ONSEN*, we crossed 13–7 and 19–4 with an *IBM2* mutant (*ibm2*). The resultant F2 progeny was genotyped at *IBM2*, *NRPD1*, and the *ONSEN* insertion in *ABI5* (*13–7*) and in *ABI4* (*19–4*). The F3 progenies derived from the genotyped parents were then exposed to ABA for analysis of ABA-insensitivity. For the F3 progeny of 13–7, an ABA-insensitive phenotype was observed 87 out of 100 seedlings in *13–7 ibm2*, although *13–7* and *13–7 nrpd1* were ABA sensitive (Fig. 4a). In contrast, the F3 progeny of 19–4 exhibited an ABA-insensitive phenotype in *19–4* (96 out of 99 seedlings), *19–4 ibm2* (97 out of 100 seedlings), and *19–4 nrpd1* (90 out of 100 seedlings) (Fig. 4a). To confirm a relationship between the phenotype and the transcription of ABA-responsive genes, we used quantitative real-time PCR to analyze transcript levels in the F3 progenies. We found that *ONSEN* insertions disrupted *ABI4* transcripts in *19–4*, *19–4 ibm2*, and *19–4 nrpd1* (Fig. 4b), while *ABI5* transcript levels were significantly recovered in *13–7* and *13–7 nrpd1* (Fig. 4b).

**Figure 5:**
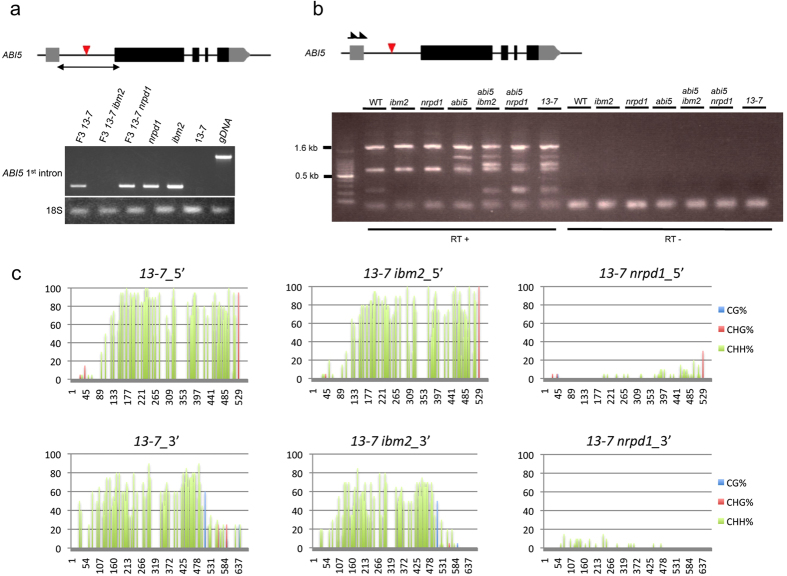
RNA expression at the *ONSEN* insertion and DNA methylation of the 5′ and 3′ flanking regions of the *ONSEN* insertion. (**a**) RT-PCR analyses of transcripts for *ABI5*. A PCR product of the *ONSEN* insertion site was not amplified in the *13–7 ibm2* and 13–7 parent line because of transcription elongation defects. In contrast, the first intron was spliced out in *13–7* and *13–7 nrpd1* of F3, similarly to the wild type. *ABI5* 1^st^ intron; the first intron that *ONSEN* was inserted in 13–7. 18S; 18S rRNA gene. Genomic DNA (gDNA) was used as a template. The red arrowhead indicates an *ONSEN* insertion. (**b**) A 3′ Rapid Amplification of cDNA Ends (RACE) of *ABI5* in the F3 progeny. The red arrowhead indicates an *ONSEN* insertion and black arrows indicate primers used in 3′ RACE. (**c**) Bisulfite sequence analysis of 5′ and 3′ flanking regions of the *ONSEN* insertion in F3 progenies (derived from the progeny of a cross between 13–7 and an *ibm2*). *13–7*: F3 progeny that contains an *ONSEN* insertion in *ABI5*. *13–7 ibm2*: F3 progeny that contains an *ONSEN* insertion in *ABI5* and a mutation in *IBM2*. *13–7 nrpd1*: F3 progeny that contains an *ONSEN* insertion in *ABI5* and a mutation in *NRPD1*.

Next, we used reverse-transcription PCR to amplify *ONSEN*-inserted regions in the F3 progeny and detect RNA expression. We found that the first intron was spliced out in *13–7* and *13–7 nrpd1*, similar to the wild type, but the expected product was not amplified in *13–7 ibm2* (Fig. 5a). To reveal whether transcription or splicing was affected in the insertion line, we conducted 3′ Rapid Amplification of cDNA Ends (RACE) of *ABI5* in the F3 progeny. The results showed that proportions of shorter forms of transcript variants were increased in *13–7 ibm2* (Fig. 5b). These results suggest that IBM2 successfully promoted RNA expression on the *ONSEN*-inserted regions in *13–7 nrpd1* and *13–7*, but in *13–7 ibm2*, *ONSEN* insertions may cause transcription elongation and/or splicing defects.

Finally, we examined the participation of DNA methylation in the epigenetic regulation of *ONSEN* insertion into *ABI5*. We observed an increase in methylation of the 500-bp 5′ and 3′ regions of the *ONSEN* insertion when 13–7 was crossed with *ibm2* mutants. Interestingly, the methylation level of the F1 between 13–7 and *nrpd1* showed a re-establishment of cytosine methylation at CHH (where H = A, T, or C) sites (see Supplementary Fig. S8). The re-establishment of cytosine methylation might responsible for the unexpected segregation of ABA insensitive progeny by the cross of 13–7 with *nrpd1*. The presence of CHH methylation in an *nrpd1* background suggests other DNA methylation pathways to establish the de novo DNA methylation besides the canonical RdDM. In the F3 progeny of 13–7, cytosine methylation at CHH sites in both *13–7 ibm2* and *13–7* were similar, whereas methylation was significantly reduced in *13–7 nrpd1* (Fig. 5c). These results suggest that the IBM2-mediated transcriptional recovery of *ABI5* and related ABA-sensitive phenotypes in the F3 progeny were independent of epigenetic regulation through the RdDM pathway.

### Heat-activation of the intragenic insertion of *ONSEN*

**Figure 6:**
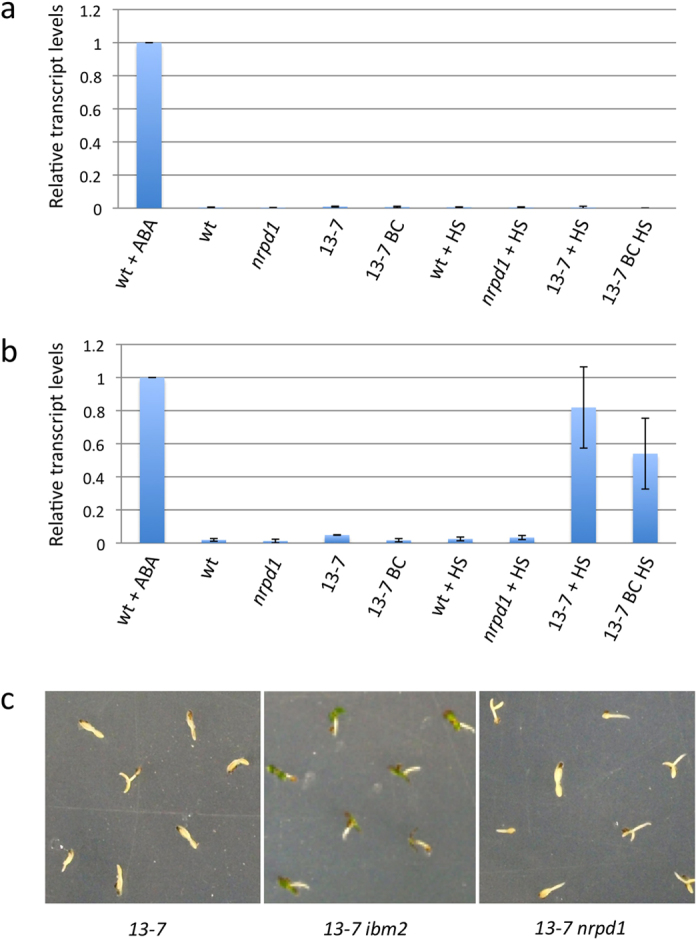
Transcriptional state of *ABI5* and ABA sensitivity with heat stress. Relative transcript level of *ABI5* without ABA stress was analyzed on the *ONSEN* inserted region (**a**) and on the downstream of the insertion (**b**). Values are relative to ABA-stressed wild-type seedlings. wt; wild-type seedlings, *nrpd1*; *nrpd1* mutant, 13–7; parental 13–7 line, 13–7 BC; F3 progeny that contains an *ONSEN* insertion in *ABI5*. (**c**) ABA-insensitive phenotype of *ABI5* in the F3 progeny subjected to heat stress. *13–7*: F3 progeny that contains an *ONSEN* insertion in *ABI5*. *13–7 ibm2*: F3 progeny that contains an *ONSEN* insertion in *ABI5* and a mutation in *IBM2*. *13–7 nrpd1*: F3 progeny that contains an *ONSEN* insertion in *ABI5* and a mutation in *NRPD1*.

It has previously been shown that new *ONSEN* insertions are able to activate genes in close proximity under heat stress that renders genes sensitive to environmental stimuli^14^. To understand the effect of the *ONSEN* transcript, we analyzed the transcript level of *ABI5* on the *ONSEN* inserted region and downstream of *ONSEN* insertion in the 13–7 line. The transcript of *ABI5* on the inserted region was not detected under heat-stress conditions (Fig. 6a). However, even without ABA stress, the *ABI5* transcript level on the downstream of the *ONSEN* insertion was upregulated under heat-stress conditions (Fig. 6b). We also analyzed the transcript level of *ABI5* on the re-activated backcross *ONSEN* insertion line of *ABI5* in the F3 progeny (*13–7 IBM2 NRPD1)*. The transcript level of *ABI5* on the downstream of *ONSEN* insertion was also upregulated under heat-stress conditions, although the transcript was not detected on the inserted region (Fig. 6a,b). To determine whether the *ONSEN* transcript confers adaptive advantages to overcome ABA stress, we analyzed the ABA sensitivity of the *ONSEN* insertion line of *ABI5* in the F3 progeny subject to heat stress under ABA stress. In the F3 progeny derived from 13–7, an ABA-insensitive phenotype was observed in *13–7 ibm2*, although *13–7* and *13–7 nrpd1* were ABA sensitive (Fig. 6c). The results indicated that heat stress lead to a reactivation of *ONSEN* in the inserted region; however, the upregulation upon heat-treatment might be the result of ectopic aberrant transcripts that did not affect the functional transcript of *ABI5*.

## Discussion

Our data have directly demonstrated that a stress-activated transposon can produce ABA-insensitive phenotypes in *Arabidopsis.* This outcome is supported by previous studies in other species^46^. In some rice strains, the DNA transposon, *mping*, rapidly increased in every generation^47^, and acted as an enhancer that rendered adjacent genes stress-inducible^48^. In soybean, the insertion of retrotransposon *SORE-1* into a paralog of phytochrome A resulted in photoperiod insensitivity, allowing for soybean cultivation at high latitudes, where the growing season is limited^49^. Although the ABA-insensitive phenotypes generated here suggest stress tolerance, more data is required to verify whether these plants actually experience fitness gains. However, it is possible that *ONSEN* transpositions could facilitate the occurrence of beneficial mutations and may contribute to adaptive evolution.

We also found evidence that *ONSEN* activation was controlled by an siRNA-mediated epigenetic regulation: *nrpd1* mutants with a deficient RdDM pathway exhibited increased *ONSEN* heat activation, and a transgenerational transposition was observed in these mutants. This finding suggests the involvement of DNA demethylation in *ONSEN* activation, similar to findings in maize demonstrating that temperature stress results in the selective demethylation of transposons^50^. However, heat-activated *ONSEN* transcription is independent of demethylation^14^,^41^. We thus propose that siRNA-mediated regulation of *ONSEN* may occur at levels other than transcription. For example, the epigenetic activation of *Athila* retrotransposons in *Arabidopsis* produces siRNA854, which regulates the formation of a stress granule-related protein on post-transcriptional and translational levels^51^. More research is required before we can fully understand the precise mechanism of siRNA-mediated *ONSEN* regulation in *Arabidopsis*.

New *ONSEN* transpositions occurred in euchromatic regions in *nrpd1* mutants. The target site preference of transposons has been reported in other plants^48^,^52^,^53^,^54^, including *Arabidopsis*^55^. The *ONSEN* target genes have on average a smaller intron compared with the genome-wide distribution. Genes with short introns minimize the cost of transcription and other molecular processes such as splicing^56^. *ONSEN* might select a target gene by splicing efficiency. Since the mutations in the RdDM components affect DNA methylation in euchromatin^57^,^58^, epigenetic modifications on the *ONSEN* target site may also be important for integration-site preferences. Unfortunately, the technical difficulties of isolating and analyzing single *ONSEN*-activated cells prevented us from obtaining more data on site preference mechanisms.

The insertion of *ONSEN* into ABA-responsive genes suggests a role for the transposon in influencing adaptive stress responses, as ABA plays a pivotal role in the latter^59^. We demonstrated salt tolerance in the *ONSEN* inserted lines that indicated the adaptation to environmental stress. Although more research is needed to investigate the actual stress responses of an *ONSEN*-integrated population, data available in maize indicates that TEs are involved in the regulation of gene responses to abiotic stress^60^.

**Figure 7:**
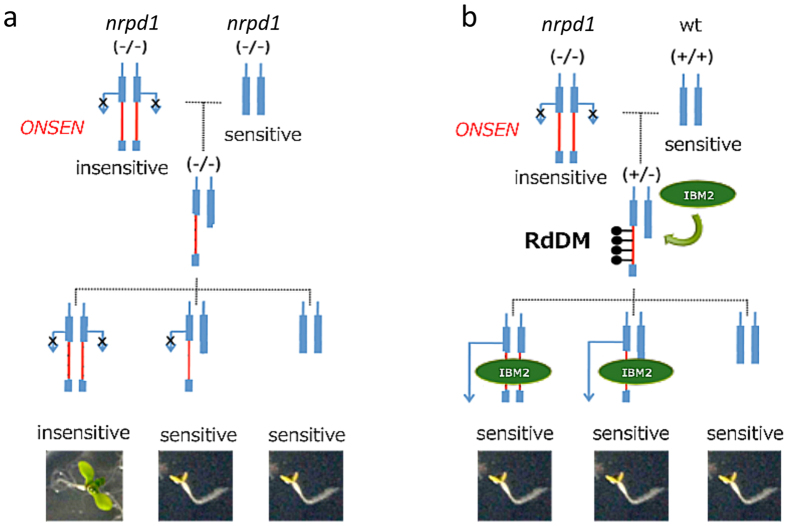
A model of ABA response in an *ONSEN*-integrated mutant. *ONSEN* is inserted into an intron of an ABA-responsive gene. (**a**) An ABA-insensitive mutant is crossed with *nrpd1*. The ABA-insensitive phenotype appears in an F2 segregant that is homozygous on the *ONSEN* insertion locus. (**b**) An ABA-insensitive mutant is crossed with a wild-type plant. IBM2 is recruited on the *ONSEN* insertion locus by RdDM, and *ONSEN* insertion is masked in the F1 and F2 segregants, resulting in ABA-sensitive phenotypes.

Finally, self-fertilization of ABA-insensitive mutants resulted in progenies that inherited ABA-insensitivity (see Supplementary Fig. S5), while crossing mutants with the wild type recovered ABA sensitivity (see Supplementary Fig. S6). We observed an increase in methylation when ABA-insensitive mutants were crossed with *ibm2* mutants (see Supplementary Fig. S8). Together, these results suggest that an NRPD1-heterozygous F1 hybrid could establish an epigenetic state that recruits IBM2 on the *ONSEN* insertion, which could be maintained in the F2 progenies independent of NRPD1, to promote proper splicing of the intron harboring *ONSEN* (Fig. 7). Consistent with this hypothesis, the loss of heterochromatic epigenetic modifications in intronic TEs affects the transcription of associated genes^31^, as we observed in *ibm2*.

The *ONSEN* insertion in *ABI5* was masked by IBM2 and the reactivation of *ONSEN* by heat-stress did not affect the functional transcription of *ABI5*. It is necessary to analyze how many stress-induced TEs acquired an adaptive advantage in the new insertion site and how many were affected by IBM2-mediated regulation of the host genome.

To conclude, the present study investigated whether the environment can directly induce genetic and epigenetic diversity for selection to act upon or whether it is simply a selective force to which existing diversity responds. We found evidence that an environmental stress-induced transposition changes the structure of the host genome and influences gene expression. The generation of mutations by *ONSEN* could play a role in the adaptive evolution of *Arabidopsis.* Future studies could expand upon these findings by investigating the biological significance (i.e., adaptive function) of epigenetic variations associated with TEs.

## Methods

### Plant material and growing conditions

The *A. thaliana* plants used in the experiments included the wild type, *nrpd1* mutants^61^, and *ibm2* mutants^30^. The seedlings were grown on Murashige and Skoog (MS) plates under continuous light at 21 °C. All wild-type and mutant plants were *A. thaliana* ecotype *Columbia*.

### SOLiD Sequencing

The sequencing library preparations and high-throughput sequencing were performed according to the manufacturer’s instructions. Total purified genomic DNA samples (1 μg) were processed into pair-endo sequencing libraries using the SOLiD fragment library construction kit (Life Technologies). After quantification by qPCR, libraries were amplified onto beads using emulsion PCR, deposited on slides, and sequenced using the SOLiD 4 sequencing system (Life Technologies). Each sample was distinguished by adding a unique “barcode” sequencing adaptor #1–16 using the SOLiD fragment library Barcode Kit (Life Technologies). Fragments were sequenced 50 and 35 bp from each end. The sequencing data from the experiment are available from the NIH short reads archive (accession GSE73097).

### ABA treatments

For ABA screening, 7-d-old seedlings were grown on MS plates containing 1 μM ABA. ABA-insensitive phenotypes of the progenies were analyzed on MS plates containing 2 μM ABA. For quantitative real-time polymerase chain reaction (qRT-PCR) and microarray analysis, RNA was extracted from 7-d-old seedlings grown on MS plates containing 2 μM ABA.

### Salt treatments

For salt-stress experiments, seedlings were grown on Murashige and Skoog (MS) plates that contained 100 mM NaCl incubated in a near vertical position under continuous light at 21 °C.

### Southern blot analysis

*Arabidopsis* genomic DNA was isolated using a Nucleon PhytoPure DNA extraction kit (GE Healthcare Life Science). Southern blots were performed following previously described protocol^62^. We detected hybridization signals in a high-SDS hybridization buffer, using a radio-labeled *ONSEN*-specific probe that was generated with the Megaprime DNA Labeling System (GE Healthcare Life Science)^63^.

### Microarray analysis

Microarrays experiments were performed using an Agilent DNA Microarray Scanner G2539A ver. C, and all procedures and data analyses followed manufacturer protocol, except where noted. We used three biological replicates in the experiments. cRNA was labeled using a Low Input Quick Amp Labeling Kit (Agilent) and hybridized to an Agilent custom array platform (Design ID = 034592). cRNA probes were designed based on expression regions of the TAIR10 genome, using previous tiling-array and RNA-Seq analyses^64^,^65^,^66^.

We normalized the signals of microarray probes to the 75^th^ percentile. We then set the following criteria to determine significance in differential gene expression: 1) changes in expression level must either be greater than 1.5-fold or less than 0.67-fold, and 2) the results of a Student’s *t*-test, adjusted with a Benjamini Hochberg FDR^67^, must yield P < 0.05.

The gene expression heat map was built using the heatmap.2 package in R ver. 2.1.12 (R Core Team). Hierarchical gene clusters were built using complete linkage clustering under Euclidean distance, with Z-scores calculated from log2 normalized values. Heat map coloration was dependent on the Z-scores. *Arabidopsis* microarray expression profiling data is available in the GEO (GSE71951).

### Quantitative real-time PCR (qRT-PCR)

To analyze the gene expression of *ABI4* and *ABI5*, total RNA was extracted from 50 seedlings using TRI Reagent (Sigma T9424), following supplier protocol. Around 3–5 μg of total RNA was treated with RQ1 RNase-free DNase (Promega) and reverse-transcribed using the ReverTraAce qPCR RT Kit (TOYOBO FSQ-101). qRT-PCR was performed using the Applied Biosystems 7300 Real Time PCR System with the THUNDERBIRD SYBR qPCR Mix (TOYOBO QPS-201). Primers used for qRT-PCR are listed in Supplementary Table S6. We performed three biological replicates and determined the standard deviation of the replicates.

### Reverse transcription-PCR (RT-PCR)

RNA was isolated from 50 7-d-old seedlings with TRI Reagent (Sigma). We used the OneStep RT-PCR Kit (QIAGEN) with gene-specific primers (see Supplementary Table S6). The thermocycling profile was as follows: 30 min at 50 °C; 15 min at 95 °C; 30 cycles of 94 °C (30 s), 55 °C (30 s), and 72 °C (1 min); and 7 min at 72 °C for *ABI5* and 30 min at 50 °C; 15 min at 95 °C; 25 cycles of 94 °C (30 s), 55 °C (30 s), and 72 °C (30 s); and 7 min at 72 °C for 18srRNA. 3′ Rapid Amplification of cDNA Ends was performed using oligo dT primers followed by the first round of PCR using gene-specific primers and the first oligo-dT-specific primer (see Supplementary Table S6). Amplified fragments were diluted and further used for the second round of PCR using another gene-specific primer and a second oligo-dT-specific primer (see Supplementary Table S6).

### DNA methylation analysis

For bisulfite sequencing analysis^68^, 0.25–1 μg of heat-denatured genomic DNA in 20 μl H_2_O was incubated with 1/9 the volume of 3 M NaOH, for 20 min at 37 °C. Next, 275 μl 10 M bisulfite solution was added to the denatured DNA sample and incubated at 70 °C for 1 h. Bisulfite-treated DNA was purified and desulfonated using an EZ DNA Methylation Kit (Zymo Research), following manufacturer protocol. We used 2 μl DNA as a template in PCR. Primers for the analysis are listed in Supplementary Table S6. Twenty clones were sequenced for each region.

## Additional Information

**How to cite this article**: Ito, H. *et al.* A Stress-Activated Transposon in *Arabidopsis* Induces Transgenerational Abscisic Acid Insensitivity. *Sci. Rep.* **6**, 23181; doi: 10.1038/srep23181 (2016).

## Supplementary Material

Supplementary Information
