# Supplementary material for: A Stress-Activated Transposon in Arabidopsis Induces Transgenerational Abscisic Acid Insensitivity
Source: Sci Rep. 2016 Mar 15;6:23181. doi: 10.1038/srep23181 (PMC4791638; doi:10.1038/srep23181)
Supplement: Supplementary Information [file srep23181-s1.pdf]

A Stress-Activated Transposon in *Arabidopsis* Induces Transgenerational Abscisic Acid Insensitivity

Hidetaka Ito[1]*, Jong-Myong Kim[2], Wataru Matsunaga[1], Hidetoshi Saze[3], Akihiro Matsui[2], Takaho A. Endo[4], Yoshiko Harukawa[3], Hiroki Takagi[5], Yaegashi Hiroki[5], Yukari Masuta[1], Seiji Masuda[1], Junko Ishida[2], Maho Tanaka[2], Satoshi Takahashi[2,7], Taeko Morosawa[2], Tetsuro Toyoda[5], Tetsuji Kakutani[6], Atsushi Kato[1], Motoaki Seki[2,7,8]

[1]Faculty of Science, Hokkaido University, Sapporo 060-0810, Japan. [2]Plant Genomic Network Research Team, RIKEN Center for Sustainable Resource Science, Yokohama 230-0045, Japan. [3]Okinawa Institute of Science and Technology, Onna-son, 904-0495, Japan. [4]RIKEN IMS-RCAI, Yokohama 230-0045, Japan. Iwate Biotechnology Research Center, Kitakami 024-0003, Japan. [5]RIKEN Advanced Center for Computing and Communication, Wako 351-0198, Japan. [6]National Institute of Genetics, Mishima 411-8540, Japan. [7]Kihara Institute for Biological Research, Yokohama City University, Yokohama 244-0813, Japan. [8]Core Research for Evolutional Science and Technology, Japan Science and Technology, Kawaguchi 332-0012, Japan

*The contact information for the corresponding author:

Kita10 Nishi8, Kita-ku, Sapporo, Hokkaido

060-0810, Japan

hito@mail.sci.hokudai.ac.jp

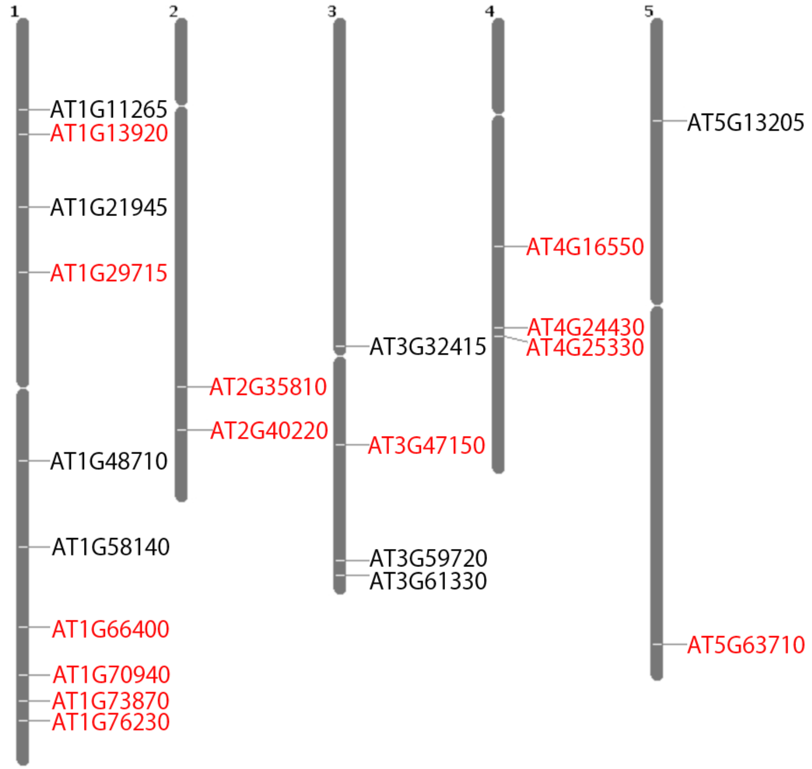

Supplementary Figure S1

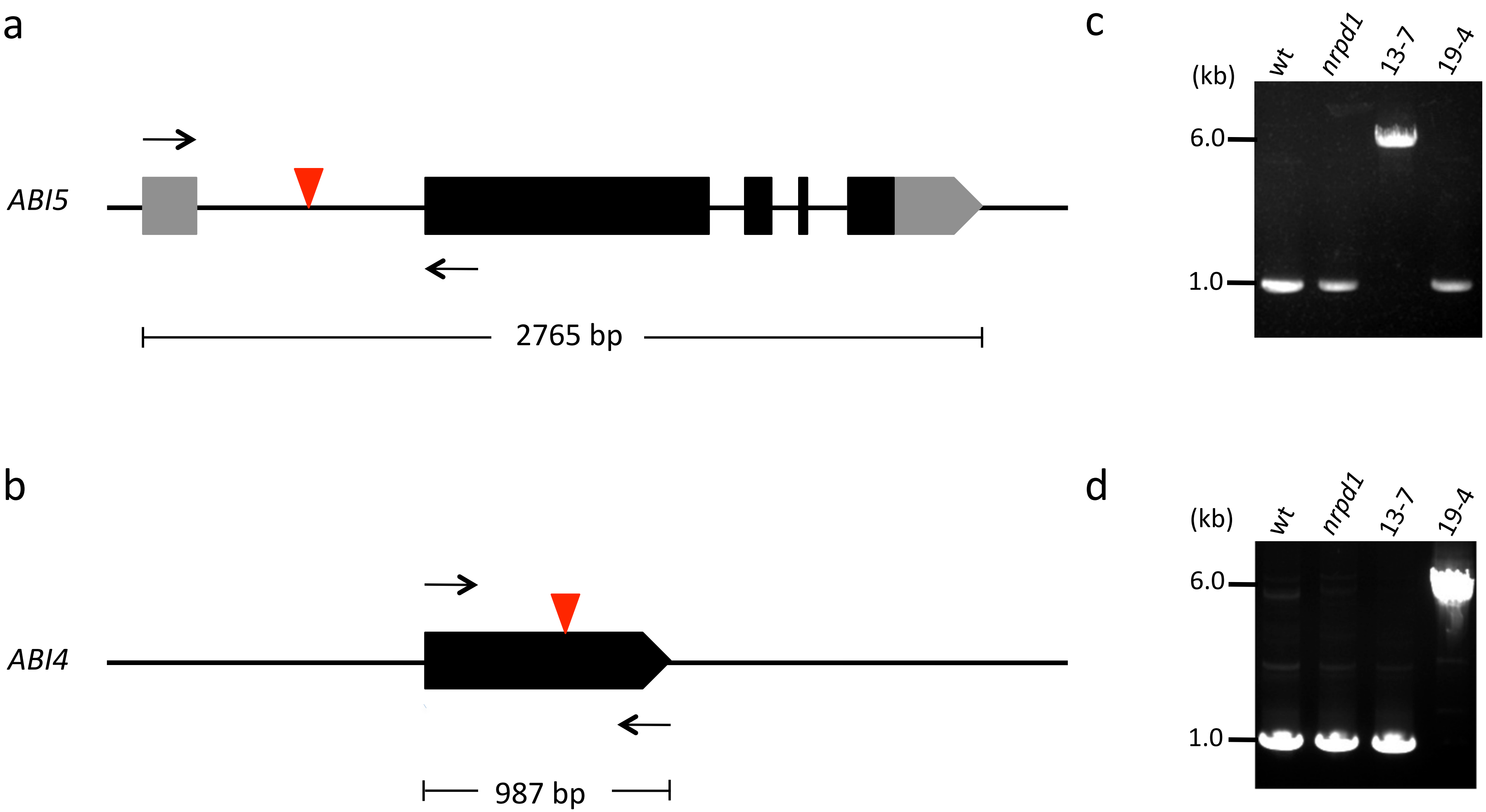

Supplementary Figure S2

```
AT5G13205  TGTTGAAAGTTAAACTTGATTTTGAATCAAGTTTAATTATTGGATCAATTATCCAATAATTAATTATGGCCAAATCCAAGTTCTAGAGTTTTCTCTAGAA
AT3G61330  TGTTGAAAGTTAAACTTGATTTTGAATCAAGTTTAATTATTGGATCAATTATCCAATAATTAATTATGGCCAAATCCAAGTTCTAGAGTTTTCTCTAGAA
AT3G59720  TGTTGAAAGTTAAACTTGATTTTGAATCAAGTTTAATTATTGGATCAATTATCCAATAATTAATTATGGCCAAATCCAAGTTCTAGAGTTTTCTCTAGAA
AT3G32415  TGTTGAAAGTTAAACTTGATTTTGAATCAAGTTTAATTATTGGATAATTTATCCAATAATTAATTATGGTCAAATCCAATTTCTAGAGTTTTCTCTAGAA
AT1G58140  TGTTGAAAGTTAAACTTGATTCAAAA----GTTTAATTATTGGATCAATTATCCAATAATTAATTATGGCCAAATCTAAGTTCTAAAGTTTTCTTTAGAA
AT1G48710  TGTTGAAAGTTAAACTTGATTTTGAATCAAGTTTAATTATTGGATCAATTATCCAATAATTAATTATGGTCAAATCCAAGTTCTAGAGTTTTCTCTAGAA
AT1G21945  TGTTGAAAGTTAAACTTGATTTTGAATCAAGTTTAATTATTGGATCAATTATCCAATAATTAATTATGGCCAAATTCAAGTTTTAGAATTTTCTCTAGAA
AT1G11265  TGTTGAAAGTTAAACTTGATTTTGAATCAAGTTTAATTATTGGATCAATTATCCAATAATTAATTATGGCCAAATCCAAGTTCTAGAGTTTTCTCTAGAA
13-7       TGTTGAAAGTTAAACTTGATTTTGAATCAAGTTTAATTATTGGATCAATTATCCAATAATTAATTATGGCCAAATCCAAGTTCTAGAGTTTTCTCTAGAA
19-4       TGTTGAAAGTTAAACTTGATTTTGAATCAAGTTTAATTATTGGATCAATTATCCAATAATTAATTATGGCCAAATCCAAGTTCTAGAGTTTTCTCTAGAA

AT5G13205  ATATCATCATTTCCACCTCCTTAAAAGATTCTAGAAATTTTCTAGAATCATCTTCCACCTCCTTAAACATAAAAAATCTAGATACTCTAATAGAATAATCT
AT3G61330  ATATCATCATTTCCACCTCCTTAAAAGATTCTAGAAATTTTCTAGAATCATCTTCCACCTCCTTAAACATAAAAAATCTAGATACTCTAATAGAATAATCT
AT3G59720  ATATCATCATTTCCACCTCCTTAAAGGATTCTAGAAATTTTCTAGAATCATCTTCCACCTCCTTAAACATAAAAAATCTAGATACTCTAATAGAATAATCT
AT3G32415  ATATCATCATTTCCACCTCCTTAAAAGATTCTAGAAATTTTCTAGAATCATCTTCCACCTCCTTAAACATAAAAAATCTAGATACTCTAATAGAATAATCT
AT1G58140  ATATCATCATTTCCATCTCCTTAAAAGATTCTAGAAATTTTCTACAATCATCTTCCACCTCCTTAAACATAAAAAATCTAGATACTTTAATAGAATAATCT
AT1G48710  ATATCATCATTTCCACCTCCTTAAAAGATTCTAGAAATTTTCTAGAATCATCTTCCACCTCCTTAAACATAAAAAATCTAGATACTCTAATAGAATAATCT
AT1G21945  ATATCATCATTTCCACCTCCTTAAAAGATTCTAGAAATTTTGTAGAATCATCTTCCACCTCCTTAAACATAAAAAATCTAGATACTCTAATAGAATAATCT
AT1G11265  ATATCATCATTTCCACCTCCTTAAAAGATTCTAGAAATTTTCTAGAATCATCTTCCACCTCCTTAAACATAAAAAATCTAGATACTCTAATAGAATAATCT
13-7       ATATCATCATTTCCACCTCCTTAAAAGATTCTAGAAATTTTCTAGAATCATCTTCCACCTCCTTAAACATAAAAAATCTAGATACTCTAATAGAATAATCT
19-4       ATATCATCATTTCCACCTCCTTAAAAGATTCTAGAAATTTTCTAGAATCATCTTCCACCTCCTTAAACATAAAAAATCTAGATACTCTAATAGAATAATCT

AT5G13205  AGATACTCTAATAGAATAATCTAGATAATTTGAATAATGTAATCTAGATCTTATGTAAGAACTCTCTAGACTTAGGATTAAAATATTTTAGATATTTTGT
AT3G61330  AGATA----------------------ATTTGAATAATGTAATCTAGATCTTATGTAAGAACTCTCTAGACTTAGGATTAAAATATTTTAGATATTTTGT
AT3G59720  AGATA----------------------ATTTGAATAATGTAATCTAGATCTTATGTAAGAACTCTCTAGACTTAAGATTAAAATATTTTAGATATTTTGT
AT3G32415  AGATA----------------------ATTTGAATAATGTAATCTAGATCTTATGTAAGAACTCTCTAGACTTAGGATTAAAATATTTTAGATATTTTGT
AT1G58140  AGATA----------------------ATTTGAATAATGTAATCTAGATCTTATATAAGAACTCTCTAGATTTAGGATTAAAATATTTTAGATATTTTGT
AT1G48710  AGATA----------------------ATTTGAATAATGTAATCTAGATCTTATGTAAGAACTCTCTAGACTTAGGATTAAAATATTTTAGATATTTTGT
AT1G21945  AGATA----------------------ATTTGAATAATGTAATCTAGATCTTATATAAGAACTCTCTAGACTTACGATTAAAATATTTTAGATATTTTGT
AT1G11265  AGATA----------------------ATTTGAATAATGTAATCTAGATCTTATGTAAGAACTCTCTAGACTTAGGATTAAAATATTTTAGATATTTTGT
13-7       AGATA----------------------ATTTGAATAATGTAATCTAGATCTTATGTAAGAACTCTCTAGACTTAGGATTAAAATATTTTAGATATTTTGT
19-4       AGATA----------------------ATTTGAATAATGTAATCTAGATCTTATGTAAGAACTCTCTAGACTTAGGATTAAAATATTTTAGATATTTTGT

AT5G13205  AGTTTGGAGGCTATAAATACCTCCTCCCCCTCTCAAATGTTGCAATGTTGTGAAGTTGTATTCAAAGTTTAAAGCAAAGTAATAAAAGTTCTATTTCCTA
AT3G61330  AGTTTGGAGGCTATAAATACCTCCTCCCCCTCTCAAATGTTGCAATGTTGTGAAGTTGTATTCAA-GTTTAAAGCAAAGTAATAAAAGTTCTATTTCCTA
AT3G59720  AGTTTGGAGGCTATAAATACCTCCTCCCCCTCTCAAATGTTGTAATGTTGTGAAGTTGTATTCAA-GTTTAAAGCAAAGTAATAAAAGTTCTATTTCCTA
AT3G32415  AGTTTGGAGGCTATAAATACCTCCTCCCCCTCTCAAATGTTGTAAGGTTGTGAAGTTGTCTTCAA-GTTTAAAGCAAAGTAATAAAAGTTCTATTTCCTA
AT1G58140  AGTTTGGAGGCTATAAATACCTCCTCCCCTTCTCAAATGTTGTAATGTTGTGAAGTTGTATTCAA-GTTTAAAGCAAAGTAATAAAAGTTCTATTTCCTA
AT1G48710  AGTTTGGAGGCTATAAATACTTCCTCCCCCTCTCAAATGTTGCAATGTTGTGAAGTTGTATTCAA-GTTTAAAGCAAAGTAATAAAAGTTCTATTTCCTA
AT1G21945  AGTTTGGAGGCTATAAATACCTCCTCCTCCTCTCAAATGTTGCAATGTTGTGAAGTTGTCTTCAA-GTTTAAAGCAAAGTAATTAAAGTTCTATTTCCAA
AT1G11265  AGTTTGGAGGCTATAAATACCTCCTCCCCCTCTCAAATGTTGCAATGTTGTGAAGTTGTATTCAA-GTTTAAAGCAAAGTAATAAAAGTTCTATTTCCTA
13-7       AGTTTGGAGGCTATAAATACCTCCTCCCCCTCTCAAATGTTGCAATGTTGTGAAGTTGTATTCAA-GTTTAAAGCAAAGTAATAAAAGTTCTATTTCCTA
19-4       AGTTTGGAGGCTATAAATACCTCCTCCCCCTCTCAAATGTTGCAATGTTGTGAAGTTGTATTCAA-GTTTAAAGCAAAGTAATAAAAGTTCTATTTCCTA

AT5G13205  AAAAACTCTCTCAAAACACTTAAACACTTTCTCCATTACCTCTAAAAGAATTTTACTCTAACAAAGTGGTATCAGAGCTTGAAGATCCTAAAGATGGCGA
AT3G61330  AAAAACTCTCTCAAAACACTTAAACACTTTCTCCATTACCTCTAAAAGAATTTTACTCTAACAAAGTGGTATCAGAGCTTGAAGATCCTAAAGATGGCGA
AT3G59720  AAAAACTCTCTCAAAACACTTAAACACTTTCTCCATTACCTCTAAAAGAATTTTACTCTAACAAAGTGGTATCAGAGCTTGAAGATCCTAAAGATGGCGA
AT3G32415  AAAAACTCTCTCAAAACACTTTAACACTTTCTCCATTACCTCTAAAAGAATTTTACTCTAACAAAGTGGTATCAGAGCTTGAAGATCCTAAAGATGGCGA
AT1G58140  AAAAACTCTCTCAAAACACTTAAACACTTTCTCCATTACCTCTAAAAGAATTTTACTCTAACAAAGTGGTATCAGAGCTTGAAGATCCTAAAGATGGCGA
AT1G48710  AAAAACTCTCTCAAAACACTTAAACACTTTCTCCATTACCTCTAAAAGAATTTTACTCTAACAAAGTGGTATCAGAGCTTGAAGATCCTAAAGATGGCGA
AT1G21945  AAAAACTCTCTCAAAACACTTAAACACTTTCTCCATTACCTCTAAAATAATTTTACTCTAACAAAGTGGTAGCAGAACTTGAAGATCCTAAAGATGGCGA
AT1G11265  AAAAACTCTCTCAAAACACTTAAACACTTTCTCCATTACCTCTAAAAGAATTTTACTCTAACAAAGTGGTATCAGAGCTTGAAGATCCTAAAGATGGCGA
13-7       AAAAACTCTCTCAAAACACTTAAACACTTTCTCCATTACCTCTAAAAGAATTTTACTCTAACAAAGTGGTATCAGAGCTTGAAGATCCTAAAGATGGCGA
19-4       AAAAACTCTCTCAAAACACTTAAACACTTTCTCCATTACCTCTAAAAGAATTTTACTCTAACAAAGTGGTATCAGAGCTTGAAGATCCTAAAGATGGCGA

AT5G13205  GTAACAATGTTCCCTTCCAAGTCCCGGTGCTCACAAAGAGCAACTATGATAATTGGAGTCTACGAATGAAGGCTATCCTAGGAGCACATGACGTGTGGGA
AT3G61330  GTAACAATGTTCCCTTCCAAGTCCCGGTGCTCACAAAGAGCAACTATGATAATTGGAGTCTACGAATGAAGGCTATCCTAGGAGCACATGACGTGTGGGA
AT3G59720  GTAACAATGTTCCCTTCCAAGTCCCGGTGCTCACAAAGAGCAACTATGATAATTGGAGTCTACGAATGAAGGCTATCCTAGGAGCACATGACGTGTGGGA
AT3G32415  GTAACAATGTTCCCTTCCATGTCCCAGTGCTCACAAAGAGCAACTATGATAATTGGAGTCTACGAATGAAGGCTATCCTAGGAGCACATGACGTGTGGGA
AT1G58140  GTAATAATGTTCCCTTCCAAGTCCCGGTGCTCACAAAGAGCAACTATGATAATTGGAGTCTACGAATGAAGGCTATCCTAGGAGCACATGACGTGTGGGA
AT1G48710  GTAACAATGTTCCCTTCCAAGTCCCGGTGCTCACAAAGAGCAACTATGATAATTGGAGTCTACGAATGAAGGCTATCCTAGGAGCACATGACGTGTGGGA
AT1G21945  GTAACAATGTTCCCTTCCAAGTCCCGGTGCTCACAAAGAGCAACTATGATAATTGGAGTCTACAAATGAAGGCTATCCTAGGAGCACATGACGTGTGGGA
AT1G11265  GTAATAATGTTCCCTTCCAAGTCCCGGTGCTCACAAAGAGCAACTATGATAATTGGAGTCTACGAATGAAGGCTATCCTAGGAGCACATGACGTGTGGGA
13-7       GTAATAATGTTCCCTTCCAAGTCCCGGTGCTCACAAAGAGCAACTATGATAATTGGAGTCTACGAATGAAGGCTATCCTAGGAGCACATGACGTGTGGGA
19-4       GTAACAATGTTCCCTTCCAAGTCCCGGTGCTCACAAAGAGCAACTATGATAATTGGAGTCTACGAATGAAGGCTATCCTAGGAGCACATGACGTGTGGGA

AT5G13205  GATAGTCGAGAAAGGTTTCATTGAACCGGAGAATGAAGGTAGTCTTTCTCAAATTCAAAAAGATGGTTTGAGAGACTCAAGAAAGAGAGACAAGAAAGCT
AT3G61330  GATAGTTGAGAAAGGTTTCATTGAACCGGAGAATGAAGGTAGTCTTTCTCAAACTCAAAAAGATGGTTTGAGAGACTCAAGAAAGAGAGACAAGAAAGCT
AT3G59720  GATAGTCGAGAAAGGTTTCATTGAACCGGAGAATGAAGGTAGTCTTTCTCAAACTCAAAAAGATGGTTTGAGAGACTCAAGAAAGAGAGACAAGAAAGCT
AT3G32415  GATAGTCGAGAAAGGTTTCATTGAACCGGAGAATGAAGGTAGTCTTTCTCAAACTCAAAAGGATGGTTTGAGAGACTCAAGAAAGAGAGACAAGAAAGCT
AT1G58140  GATAGTCGAGAAAGGTTTCATTGAACCGGAGAATGAAGGTAGTCTTTCTCAAACTCAAAAAGATGGTTTGAGAGACTCAAGAAAGAGAGACAAGAAAGCT
AT1G48710  GATAGTCGAGAAAGGTTTCATTGAACCGGAGAATGAAGGTAGTCTTTCTCAAACTCAAAAAGATGGTTTGAGAGACTCAAGAAAGAGAGACAAGAAAGCT
AT1G21945  GATAGTCGAGAAAGGTTTCATTGAACCGGAGAATGAAGGTAGTCTTTCTCAAACTCAAAAGGATGGTTTGAGAGACTCAAGAAAGAGAGACAAGAAAGCT
AT1G11265  GATAGTCGAGAAAGGTTTCATTGAACCGGAGAATGAAGGTAGTCTTTCTCAAACTCAAAAAGATGGTTTGAGAGACTCAAGAAAGAGAGACAAGAAAGCT
13-7       GATAGTCGAGAAAGGTTTCATTGAACCGGAGAATGAAGGTAGTCTTTCTCAAACTCAAAAAGATGGTTTGAGAGACTCAAGAAAGAGAGACAAGAAAGCT
19-4       GATAGTTGAGAAAGGTTTCATTGAACCGGAGAATGAAGGTAGTCTTTCTCAAACTCAAAAAGATGGTTTGAGAGACTCAAGAAAGAGAGACAAGAAAGCT
```

Supplementary Figure S3

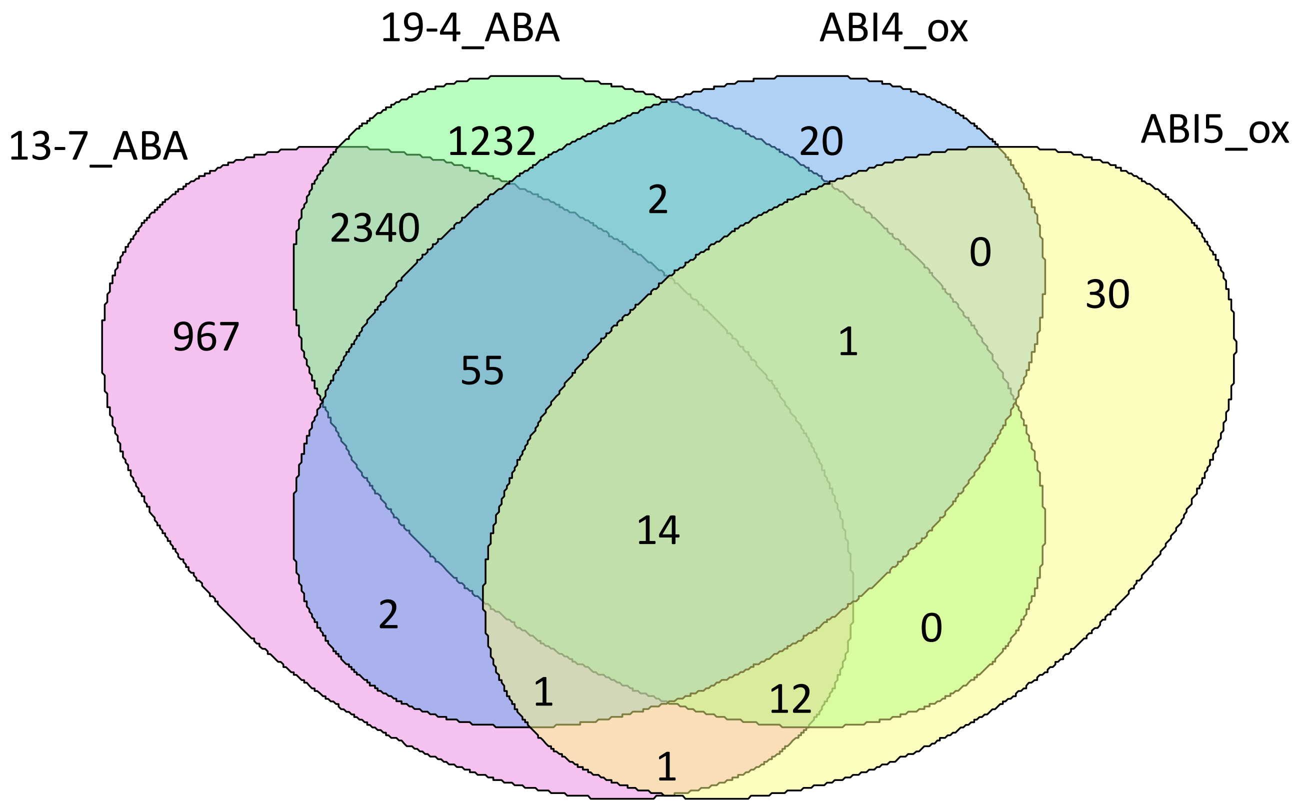

Supplementary Figure S4

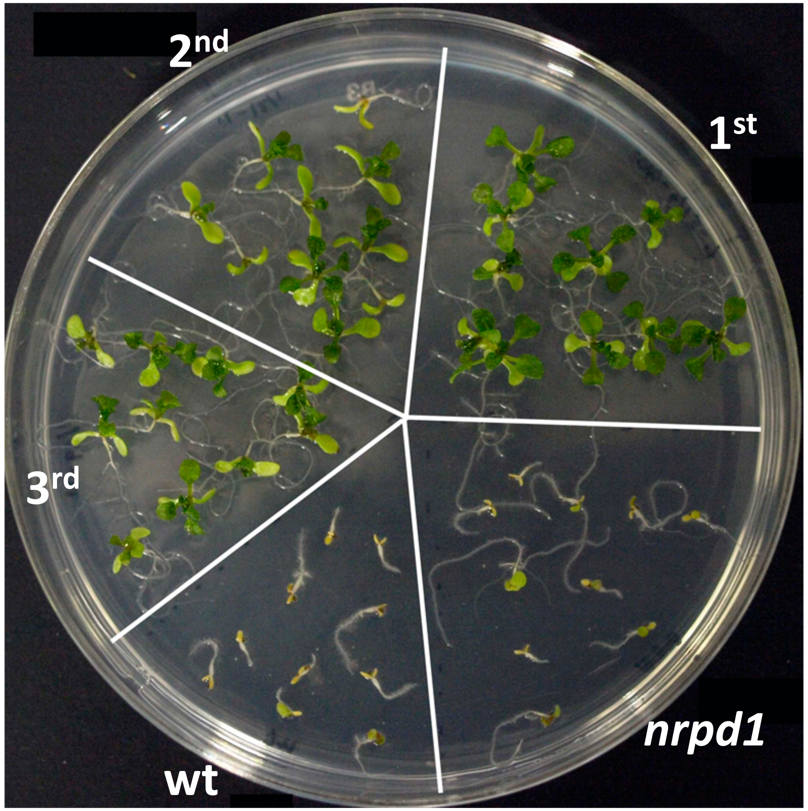

Supplementary Figure S5

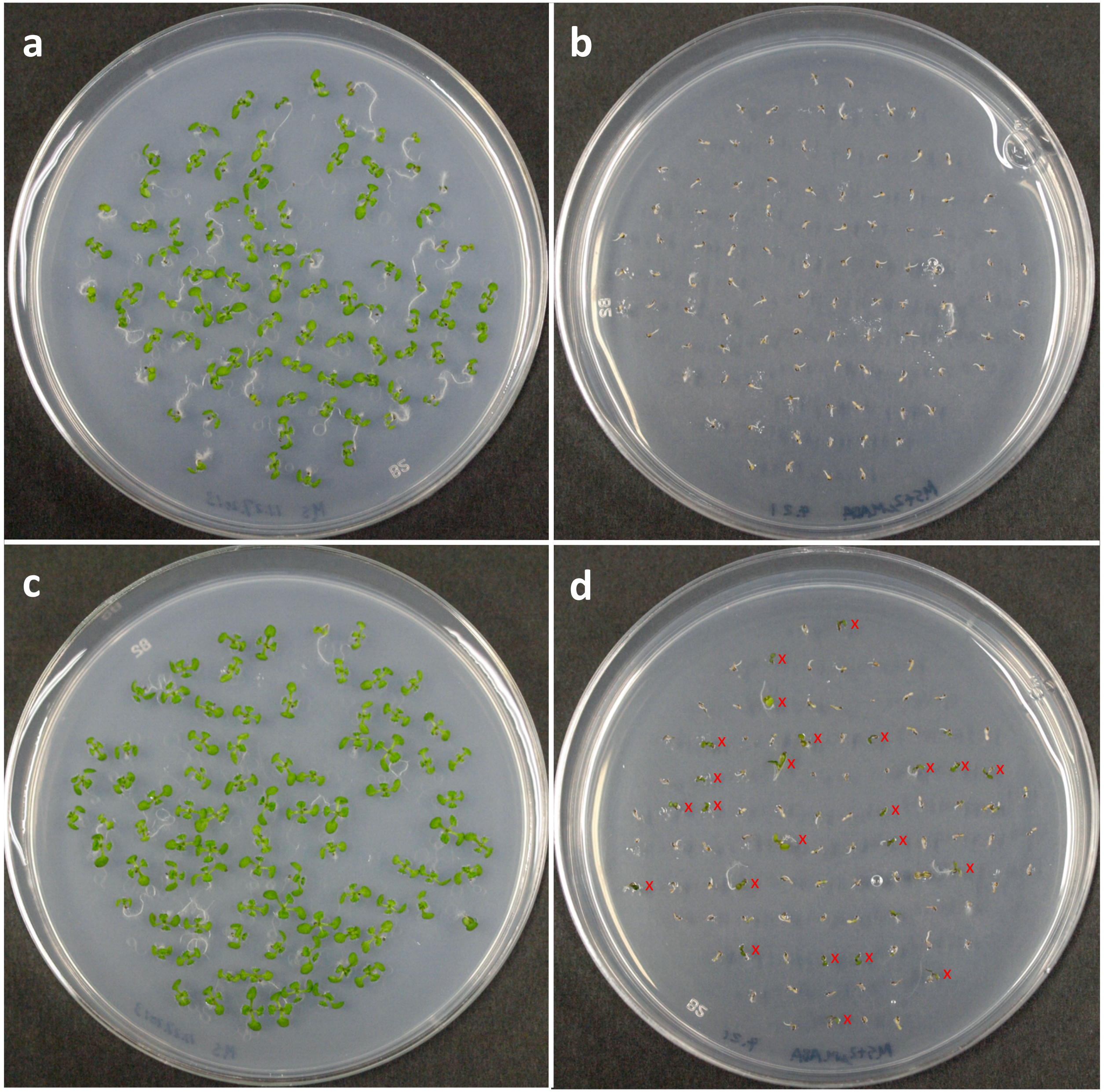

Supplementary Figure S6

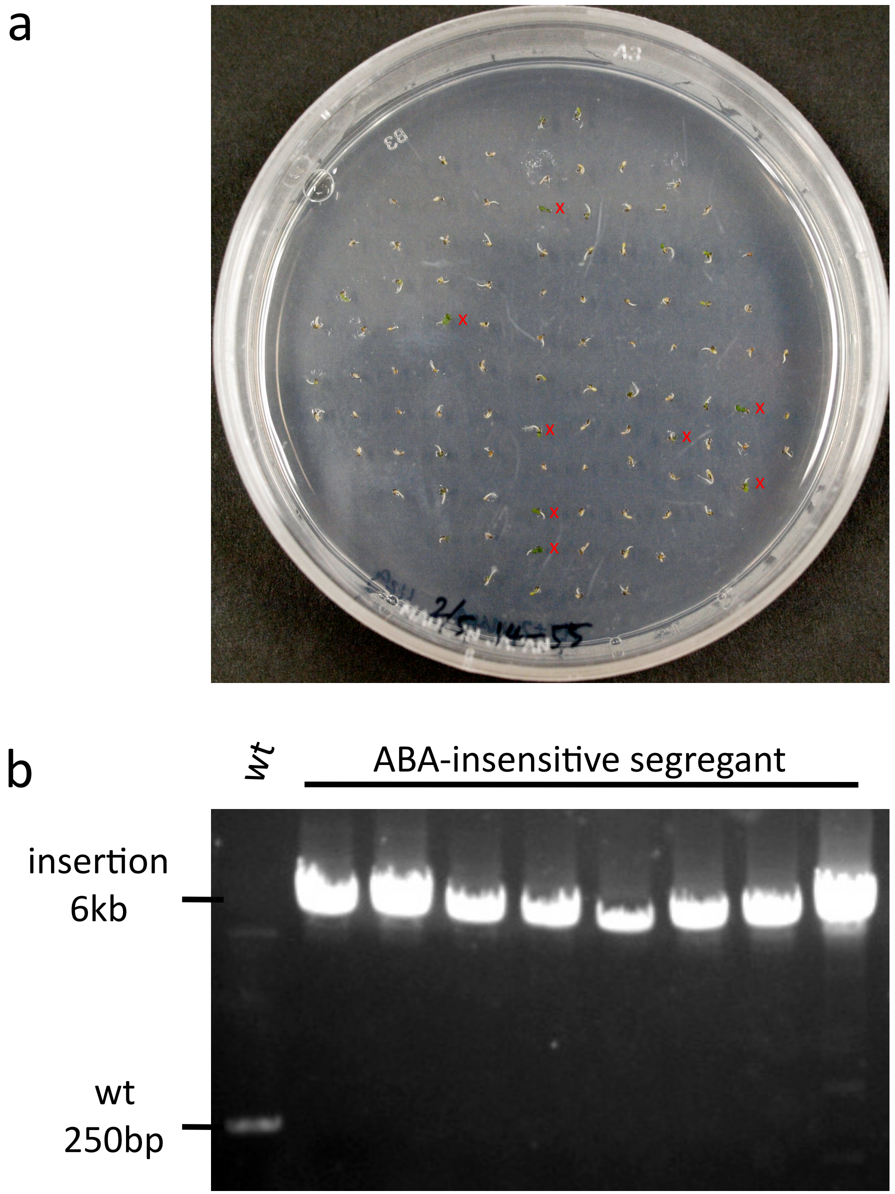

Supplementary Figure S7

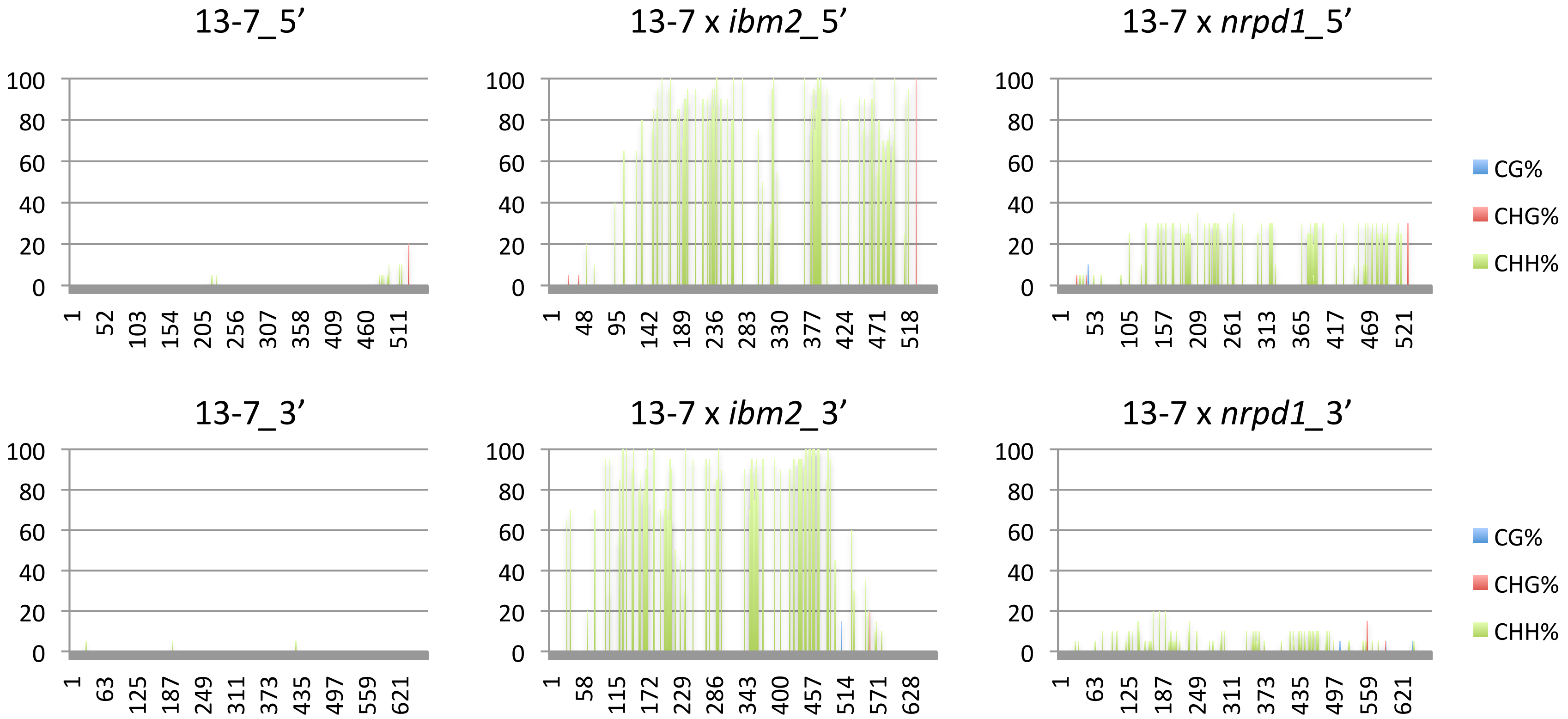

Supplementary Figure S8

Supplementary Figure S1.

Mapping of *ONSEN*-integrated genes in 19-4. Thirteen new insertions were identified within genes. The loci names with red letters indicate the new insertions and the black letters indicate the original eight *ONSEN* copies.

Supplementary Figure S2.

An *ONSEN* insertion in 13-7 and 19-4. (a, b) *ONSEN* insertion in the first intron of *ABI5* in 13-7 (a) and an exon of *ABI4* in 19-4 (b). The red arrowheads indicate the *ONSEN* insertion loci. Black arrows indicate the primer sites used for PCR. (c, d) PCR amplification of *ONSEN* in 13-7 (c) and in 19-4 (d). wt, wild type; *nrpd1*, original *nrpd1* mutant line.

Supplementary Figure S3. A 700-bp sequence of the LTR and the gene-coding region of *ONSEN*. The first eight gene IDs refer to the original, endogenous *ONSEN* copies. 13-7 and 19-4 represent the sequences of newly inserted *ONSEN* copies within *ABI5* and *ABI4*, respectively. The nucleotides marked blue and red color indicated the polymorphisms to make the identification of 13-7 and 19-4 respectively.

Supplementary Figure S4. Venn diagram of differentially expressed genes in 13-7 and 19-4 lines from our microarray analysis, and ABI4- and ABI5-direct target genes in ABI4- and ABI5-overexpressed seedlings from public microarray data. Microarray analysis identified significant differentially expressed genes of 13-7 and 19-4, compared with parental *nrpd1* under ABA treatment (Expression change was 1.5-fold or 0.67-fold, FDR < 0.05). The ABI4- and ABI5-direct target genes were used from the Supplementary material 2 of previous report (37).

Supplementary Figure S5. An ABA-insensitive phenotype of self-fertilized 13-7 progeny on 2 μM ABA. 1st, 2nd, 3rd: the generation of self-fertilized progeny.

Supplementary Figure S6.

Phenotypes of the F2 population derived from a single F1 progeny of a cross between an ABA-insensitive mutant (13-7 or 19-4) and a wild-type plant. (a, b) F2 phenotype

(cross between 13-7 and wild type) growing under normal conditions (a) and under 2 μM ABA (b). (c, d) F2 phenotype (cross between 19-4 and wild type) growing under normal conditions (c) and under 2 μM ABA (d). Red crosses indicate ABA-insensitive phenotypes (24 out of 100 seedlings).

Supplementary Figure S7.
Phenotypes and *ONSEN* insertion of the F2 population derived from a single F1 progeny of a cross between 13-7 and *nrpd1*. (a) Eight seedlings showed ABA-insensitive phenotypes growing under 2 μM ABA (red crosses). (b) *ONSEN* insertion was detected by PCR. PCR primers were designed to amplify a 250 bp region of the first intron of *ABI5*. The eight ABA-insensitive segregant were correlated with the marked seedlings in (a).

Supplementary Figure S8. Bisulfite sequence analysis of 5′ and 3′ flanking regions of the *ONSEN* insertion in 13-7 and the F1 hybrid between 13-7 and an *IBM2* mutant or between 13-7 and a *NRPD1* mutant.

Supplementary Table S1. Mapping of *ONSEN* new insertions in 13-7.

Chromosome	Gene	Start	End	Direction	Gene direction	Exon/Intron
1	AT1G03400	843611	843644	-	-	Intron
1	AT1G30790	10933742	10933800	+	+	Exon
1	AT1G58400	21696494	21696557	-	-	Exon
1	AT1G66050	24591439	24591487	-	+	Exon
2	AT2G02700	757522	757558	-	-	Exon
2	Centromere	3189065	3189255	-	NA	NA
2	NA	10540220	10540272	+	NA	NA
2	NA	11822642	11822706	-	NA	NA
2	AT2G36270	15207129	15207251	-	-	Intron
2	AT2G43570	18077261	18077329	+	-	Exon
3	AT3G01015	3182	3239	-	-	Exon/Intron
3	AT3G04945	1369214	1369286	+	-	Exon
3	AT3G13810	4545286	4545381	-	+	Intron
3	AT3G19500	6760170	6760193	-	-	Exon
3	AT3G25180	9168023	9168068	-	-	Exon
4	AT4G01930	838873	838932	-	-	Exon
4	AT4G23160	12131610	12131648	+	+	Exon
5	AT5G02160	426463	426503	+	+	Exon
5	NA	5347645	5347689	-	NA	NA
5	AT5G38550	15435629	15435656	-	-	Intron
5	AT5G54067	21941797	21941852	-	-	3'UTR
5	AT5G59070	23845240	23845295	+	+	Exon
5	AT5G59590	24009802	24009956	+	-	Exon
5	AT5G65310	26103267	26103351	-	-	Intron

Start and End show the mapped position of *ONSEN* reads.
Direction and Gene direction show the polarity of *ONSEN* and the inserted gene respectively. +; sense strand, -; antisense strand.
Exon/Intron show the definition of a gene region that *ONSEN* is inserted.

Supplementary Table S2. Mapping of *ONSEN* new insertions in 19-4.

Chromosome	Gene	Start	End	Direction	Gene direction	Exon/Intron
1	AT1G13920	4759156	4759344	+	+	Exon
1	AT1G29715	10389155	10389365	+	-	Exon
1	AT1G66400	24771163	24771369	+	-	Exon
1	AT1G70940	26744168	26744373	-	+	Exon
1	AT1G73870	27779109	27779240	+	+	5'UTR
1	AT1G76230	28600391	28600573	-	-	5'UTR
2	NA	2780595	2781605	+	NA	NA
2	NA	3189039	3189325	-	NA	NA
2	Centromere	3614998	3627989	+	NA	NA
2	NA	9711470	9711513	-	NA	NA
2	AT2G35810	15049384	15049570	+	+	Intron
2	AT2G40220	16796815	16797021	-	-	Exon
3	NA	11654581	11654770	-	NA	NA
3	NA	13540137	13540245	+	NA	NA
3	NA	14403089	14403901	+	NA	NA
3	AT3G47150	17358636	17358814	+	+	Exon
4	AT4G16550	9320354	9320402	+	-	Intron
4	AT4G24430	12631841	12632026	-	+	Exon/Intron
4	AT4G25330	12958068	12958209	+	+	Exon
5	AT5G63710	25502730	25502862	-	+	3'UTR
5	NA	26111680	26111847	+	NA	NA

Start and End show the mapped position of *ONSEN* reads.
Direction and Gene direction show the polarity of *ONSEN* and the inserted gene respectively. +; sense strand, -; antisense strand.
Exon/Intron show the definition of a gene region that *ONSEN* is inserted.

Supplementary Table S3. The properties of the *ONSEN* targeted genes.

Gene_id	Gene length (bp)	Exon number	Exon length (bp)	Intron length (bp)
AT1G03400.1	1444	3	1056	208
AT1G30790.1	1354	1	1200	0
AT1G58400.1	2954	3	2703	251
AT1G66050.1	3261	9	1872	1211
AT2G02700.1	1500	1	1500	0
AT2G36270.1	2765	4	1329	1045
AT2G43570.1	1240	2	834	213
AT3G01015.1	2507	8	1467	753
AT3G04945.1	419	2	231	188
AT3G13810.1	2921	3	1542	818
AT3G13810.2	3150	4	1545	1252
AT3G13810.3	3150	4	1503	1269
AT3G19500.1	1924	5	813	792
AT3G25180.1	1998	3	1548	280
AT3G25180.2	1996	2	1209	173
AT4G01930.1	1959	1	1959	0
AT4G23160.1	4703	10	3789	813
AT5G02160.1	1072	2	390	243
AT5G38550.1	2999	5	1785	925
AT5G54067.1	545	1	381	0
AT5G59070.1	2196	2	1518	575
AT5G59590.1	1667	2	1350	84
AT5G65310.1	2655	3	939	822
AT5G65310.2	1973	2	885	513
AT1G13920.1	1567	7	1038	529
AT1G66400.1	797	1	474	0
AT1G70940.1	3342	6	1923	779
AT1G73870.1	1511	2	1179	130
AT1G76230.1	683	1	390	0
AT2G35810.1	1174	3	600	316
AT2G40220.1	987	1	987	0
AT3G47150.1	1175	2	1083	92
AT4G16550.1	5813	16	2232	3581
AT4G24430.1	3058	13	1941	1017
AT4G25330.1	881	3	690	160
AT5G63710.1	3385	11	1845	1279
Average	2131.25	4.11	1325.83	564.19
Genome Average	2350.77	5.04	1110.31	965.24
FTEST	1.39E-02	7.68E-03	3.77E-02	3.58E-04
TTEST	2.72E-01	1.39E-01	7.77E-02	*1.04E-03

* Significant difference $p<0.05$

Supplementary Table S4. Comparison of the gene expressions of *ONSEN* inserted loci in wild type and *nrpd2* under heat stress.

13_7	Log2 expression changes				WT con		WT heat		WT recovery		*nrpd2* con		*nrpd2* heat		*nrpd2* recovery	
Inserted loci	WT heat	WT recovery	*nrpd2* heat	*nrpd2* recovery	average	s.d.	average	s.d.	average	s.d.	average	s.d.	average	s.d.	average	s.d.
AT1G03400	-2.4	-1.0	-2.1	-0.4	7.7	0.1	5.3	5.3	6.7	0.4	7.5	0.3	5.4	0.7	7.1	0.1
AT1G30790	0.28	0.1	0.3	0.0	2.9	0.0	3.2	3.2	3.0	0.2	3.0	0.1	3.2	0.2	3.0	0.1
AT1G58400	no probe	-	-	-	-	-	-	-	-	-	-	-	-	-	-	-
AT1G66050	no probe	-	-	-	-	-	-	-	-	-	-	-	-	-	-	-
AT2G02700	0.28	0.0	0.3	0.1	2.7	0.1	3.0	3.0	2.8	0.2	2.7	0.0	3.0	0.3	2.7	0.1
AT2G36270	0.50	0.1	0.5	-0.3	4.1	0.2	4.6	4.6	4.2	0.2	4.2	0.3	4.7	0.1	3.9	0.0
AT2G43570	-1.99	2.9	-0.5	4.5	5.9	0.7	4.0	4.0	8.9	0.6	5.3	1.9	4.8	0.4	9.9	0.5
AT3G01015	0.15	0.0	0.4	-0.2	2.6	0.1	2.8	2.8	2.7	0.0	2.7	0.1	3.1	0.3	2.6	0.1
AT3G04945	no probe	-	-	-	-	-	-	-	-	-	-	-	-	-	-	-
AT3G13810	-2.5	0.0	-1.9	0.3	5.7	0.2	3.2	3.2	5.7	0.3	5.5	0.1	3.5	0.2	5.8	0.3
AT3G19500	0.19	0.1	0.1	-0.1	3.5	0.1	3.7	3.7	3.7	0.2	3.8	0.1	3.9	0.2	3.6	0.0
AT3G25180	0.50	0.1	0.4	0.5	3.1	0.0	3.6	3.6	3.2	0.1	3.1	0.3	3.5	0.6	3.6	0.4
AT4G01930	0.18	0.2	0.0	0.0	2.7	0.1	2.9	2.9	2.8	0.1	2.8	0.1	2.9	0.2	2.8	0.1
AT4G23160	no probe	-	-	-	-	-	-	-	-	-	-	-	-	-	-	-
AT5G02160	0.39	0.8	0.4	-0.1	9.6	0.2	9.9	9.9	10.4	0.5	9.4	0.2	9.8	0.3	9.3	0.3
AT5G38550	no probe	-	-	-	-	-	-	-	-	-	-	-	-	-	-	-
AT5G54067	no probe	-	-	-	-	-	-	-	-	-	-	-	-	-	-	-
AT5G59070	0.07	0.1	0.2	0.0	3.1	0.2	3.2	3.2	3.2	0.1	3.2	0.1	3.4	0.3	3.1	0.1
AT5G59590	0.1	0.0	0.5	0.0	3.2	0.0	3.3	3.3	3.2	0.2	3.2	0.1	3.6	0.1	3.1	0.2
AT5G65310	-1.5	1.1	-1.6	0.0	4.2	0.1	2.7	2.7	5.3	0.7	4.5	0.2	2.9	0.0	4.5	0.6

19_4	Log2 expression changes				WT con		WT heat		WT recovery		*nrpd2* con		*nrpd2* heat		*nrpd2* recovery	
Inserted loci	WT heat	WT recovery	*nrpd2* heat	*nrpd2* recovery	average	s.d.	average	s.d.	average	s.d.	average	s.d.	average	s.d.	average	s.d.
AT1G13920	0.3	-0.1	0.1	-0.1	2.9	0.1	3.3	3.3	2.8	0.1	3.0	0.2	3.1	0.1	3.0	0.1
AT1G29715	no probe	-	-	-	-	-	-	-	-	-	-	-	-	-	-	-
AT1G66400	2.3	0.6	0.9	0.3	3.5	0.3	5.8	5.8	4.1	0.1	3.5	0.2	4.3	0.1	3.7	0.5
AT1G70940	-2.8	-1.0	-2.5	-0.8	8.1	0.1	5.3	5.3	7.2	0.4	7.8	0.2	5.3	0.6	7.0	0.4
AT1G73870	-3.0	-1.1	-2.9	-1.4	8.2	0.3	5.2	5.2	7.0	0.2	7.9	0.3	4.9	0.3	6.5	0.3
AT1G76230	0.3	0.1	0.3	0.1	2.3	0.0	2.5	2.5	2.4	0.0	2.3	0.2	2.6	0.1	2.4	0.1
AT2G35810	no probe	-	-	-	-	-	-	-	-	-	-	-	-	-	-	-
AT2G40220	0.1	0.0	0.2	-0.2	3.8	0.1	3.9	3.9	3.8	0.1	3.8	0.1	4.1	0.1	3.6	0.2
AT3G47150	no probe	-	-	-	-	-	-	-	-	-	-	-	-	-	-	-
AT4G16550	no probe	-	-	-	-	-	-	-	-	-	-	-	-	-	-	-
AT4G24430	-0.1	0.2	0.1	0.1	3.0	0.2	2.9	2.9	3.1	0.2	3.1	0.1	3.1	0.4	3.2	0.2
AT4G25330	0.1	-0.1	0.3	0.0	2.8	0.1	2.9	2.9	2.7	0.1	2.6	0.1	2.9	0.1	2.7	0.1
AT5G63710	-1.1	-0.5	-1.4	-0.5	5.3	0.2	4.2	4.2	4.8	0.2	5.1	0.3	3.7	0.3	4.6	0.2

Supplementary Table S5. Gene expression of *ONSEN*-inserted loci during flower development

13_7	Ratio compared with 0d												
Inserted loci	1d/0d	1.5d/0d	2d/0d	2.5d/0d	3d/0d	3.5d/0d	4d/0d	4.5d/0d	5d/0d	7d/0d	9d/0d	11d/0d	13d/0d
AT1G03400	-0.1	-0.1	-0.1	0.0	-0.1	0.0	-0.1	-0.2	-0.1	-0.3	-0.2	-0.2	-0.3
AT1G30790	0.2	-0.1	0.1	0.1	0.3	0.3	0.0	0.1	0.2	0.2	-0.9	-0.4	-0.2
AT1G58400	0.0	0.0	0.0	0.0	-0.1	-0.1	0.0	0.0	0.0	0.1	0.2	0.2	0.3
AT1G66050	0.0	-0.1	0.0	0.0	0.0	0.0	0.1	0.1	0.2	0.2	0.4	0.2	0.6
AT2G24750	0.0	0.0	0.1	0.0	0.0	0.0	0.1	0.0	0.2	0.0	0.0	0.1	0.1
AT2G02700	0.0	0.0	0.0	-0.1	0.0	0.0	0.0	0.0	0.0	0.0	0.0	0.0	0.2
AT2G36270	-0.1	-0.4	-0.6	-0.8	-0.9	-0.9	-1.0	-1.0	-1.0	-0.9	-0.9	-1.1	-1.3
AT2G43570	-0.2	0.1	0.3	0.4	0.6	0.5	0.6	0.1	0.2	-0.2	-0.5	-0.3	-0.6
AT3G01015	0.0	0.0	0.0	0.0	0.0	0.0	0.0	0.0	-0.1	-0.1	0.3	0.2	-0.1
AT3G04945	0.1	0.1	0.1	0.1	0.1	0.1	0.0	0.1	0.0	0.1	-0.1	0.1	0.2
AT3G13810	-0.1	-0.2	-0.2	-0.3	-0.3	-0.3	-0.4	-0.4	-0.4	-0.3	-0.1	0.0	-0.1
AT3G19500	0.1	0.2	0.3	0.4	0.5	0.6	0.7	0.8	0.7	1.0	1.3	0.9	0.9
AT3G25180	0.1	0.1	0.5	0.4	0.5	0.7	1.0	0.9	1.1	1.0	0.4	-0.3	-0.4
AT4G01930	0.1	0.0	0.1	0.0	-0.1	-0.1	0.0	0.1	0.1	0.0	0.2	0.2	0.2
AT4G23160	0.0	0.1	0.0	0.0	0.0	0.0	-0.1	0.0	0.0	-1.0	-0.3	0.1	-0.6
AT5G02160	0.0	0.0	0.0	0.0	0.0	0.0	-0.1	-0.1	-0.1	-0.2	-0.4	-0.4	-0.2
AT5G38550	-0.1	-0.1	-0.1	-0.1	-0.1	-0.1	0.0	-0.1	-0.1	-0.1	0.0	-0.1	-0.4
AT5G54067	0.1	0.1	0.1	0.1	0.1	0.1	0.1	0.1	0.1	0.1	0.1	-0.1	0.1
AT5G59070	-0.1	0.0	-0.1	0.0	0.1	0.1	0.3	0.3	0.4	0.3	-0.3	-0.6	-1.0
AT5G59590	0.0	0.1	0.0	0.0	0.0	0.1	0.2	0.0	-0.2	0.0	-0.6	-0.9	-0.2
AT5G65310	-0.2	-0.2	-0.2	-0.2	-0.2	-0.2	-0.2	-0.2	-0.1	0.0	-0.1	-0.2	-0.4

13_7	0d		1d		1.5d		2d		2.5d		3d		3.5d	
Inserted loci	average	s.d	average	s.d	average	s.d	average	s.d	average	s.d	average	s.d	average	s.d
AT1G03400	0.2	0.1	0.1	0.1	0.1	0.1	0.1	0.2	0.2	0.1	0.1	0.1	0.2	0.3
AT1G30790	0.2	0.1	0.3	0.1	0.1	0.1	0.2	0.3	0.3	0.1	0.5	0.3	0.4	0.0
AT1G58400	-0.1	0.1	-0.1	0.2	-0.1	0.2	-0.1	0.1	-0.1	0.2	-0.2	0.1	-0.2	0.3
AT1G66050	-0.1	0.0	-0.2	0.0	-0.2	0.1	-0.1	0.0	-0.1	0.0	-0.1	0.1	-0.1	0.0
AT2G24750	0.0	0.0	0.0	0.0	0.0	0.0	0.1	0.1	0.0	0.0	0.0	0.0	0.0	0.0
AT2G02700	0.0	0.0	0.0	0.0	0.0	0.0	0.0	0.0	-0.1	0.1	0.0	0.0	0.0	0.0
AT2G36270	0.9	0.0	0.8	0.0	0.5	0.1	0.3	0.1	0.1	0.0	0.0	0.0	0.0	0.0
AT2G43570	0.1	0.1	-0.1	0.2	0.1	0.2	0.3	0.1	0.5	0.1	0.7	0.2	0.6	0.1
AT3G01015	-0.1	0.1	-0.1	0.0	-0.1	0.1	-0.1	0.0	-0.1	0.1	-0.1	0.1	-0.2	0.0
AT3G04945	-0.1	0.2	0.0	0.0	0.0	0.0	-0.1	0.1	-0.1	0.1	0.0	0.2	-0.1	0.5
AT3G13810	0.2	0.1	0.1	0.1	0.0	0.0	-0.1	0.1	-0.1	0.1	-0.1	0.1	-0.2	0.1
AT3G19500	-0.6	0.1	-0.5	0.0	-0.5	0.0	-0.3	0.0	-0.2	0.1	-0.2	0.0	0.0	0.1
AT3G25180	-0.1	0.1	0.0	0.1	0.0	0.1	0.4	0.0	0.3	0.3	0.4	0.0	0.6	0.2
AT4G01930	-0.2	0.2	-0.1	0.1	-0.2	0.2	-0.1	0.1	-0.1	0.1	-0.3	0.2	-0.2	0.0
AT4G23160	0.0	0.1	0.0	0.0	0.1	0.1	0.0	0.0	0.0	0.0	0.0	0.0	0.0	0.0
AT5G02160	0.0	0.0	0.0	0.0	0.0	0.0	0.1	0.0	0.0	0.1	0.0	0.0	0.0	0.0
AT5G38550	0.1	0.1	0.0	0.0	0.0	0.1	-0.1	0.1	-0.1	0.1	-0.1	0.2	-0.1	0.3
AT5G54067	-0.1	0.1	0.0	0.0	0.0	0.0	0.0	0.0	0.0	0.0	0.0	0.0	0.0	0.0
AT5G59070	0.3	0.1	0.2	0.2	0.3	0.2	0.2	0.1	0.3	0.1	0.4	0.0	0.4	0.1
AT5G59590	0.3	0.1	0.3	0.1	0.3	0.1	0.2	0.0	0.3	0.1	0.3	0.1	0.4	0.1
AT5G65310	0.2	0.1	0.1	0.0	0.1	0.0	0.0	0.1	0.0	0.1	0.0	0.1	0.0	0.0

13_7	4d		4.5d		5d		7d		9d		11d		13d	
Inserted loci	average	s.d	average	s.d	average	s.d	average	s.d	average	s.d	average	s.d	average	s.d
AT1G03400	0.1	0.1	0.0	0.2	0.0	0.2	-0.2	0.1	-0.1	0.1	0.0	0.1	-0.2	0.1
AT1G30790	0.1	0.1	0.3	0.2	0.3	0.1	0.4	0.0	-0.8	0.2	-0.3	0.2	0.0	0.0
AT1G58400	-0.2	0.1	-0.1	0.2	-0.2	0.3	0.0	0.1	0.1	0.1	0.1	0.1	0.2	0.2
AT1G66050	-0.1	0.1	0.0	0.0	0.1	0.1	0.1	0.0	0.2	0.0	0.1	0.1	0.5	0.1
AT2G24750	0.1	0.1	0.0	0.0	0.2	0.4	0.0	0.0	0.0	0.0	0.1	0.2	0.1	0.2
AT2G02700	0.0	0.0	0.0	0.0	0.0	0.0	0.0	0.0	0.0	0.0	0.0	0.0	0.2	0.3
AT2G36270	-0.1	0.1	-0.1	0.1	-0.1	0.0	0.0	0.0	0.0	0.1	-0.2	0.1	-0.4	0.1
AT2G43570	0.6	0.1	0.2	0.2	0.3	0.4	-0.2	0.1	-0.4	0.2	-0.2	0.2	-0.5	0.1
AT3G01015	-0.2	0.0	-0.1	0.1	-0.2	0.1	-0.2	0.1	0.1	0.1	0.1	0.1	-0.3	0.1
AT3G04945	-0.1	0.1	0.0	0.1	-0.1	0.2	-0.1	0.1	-0.2	0.3	0.0	0.0	0.0	0.0
AT3G13810	-0.2	0.0	-0.2	0.0	-0.3	0.1	-0.2	0.0	0.1	0.1	0.2	0.1	0.1	0.1
AT3G19500	0.0	0.1	0.2	0.1	0.1	0.1	0.4	0.2	0.6	0.0	0.3	0.2	0.2	0.0
AT3G25180	0.9	0.2	0.8	0.2	1.0	0.1	0.9	0.1	0.3	0.3	-0.4	0.4	-0.5	0.1
AT4G01930	-0.2	0.2	-0.1	0.3	-0.1	0.3	-0.1	0.1	0.0	0.2	0.0	0.1	0.1	0.1
AT4G23160	-0.1	0.2	0.0	0.0	0.0	0.0	-1.0	0.1	-0.3	0.5	0.1	0.1	-0.6	0.2
AT5G02160	0.0	0.1	0.0	0.0	-0.1	0.1	-0.2	0.1	-0.4	0.1	-0.3	0.1	-0.2	0.1
AT5G38550	0.0	0.0	0.0	0.0	-0.1	0.1	0.0	0.0	0.0	0.0	0.0	0.0	-0.4	0.2
AT5G54067	0.0	0.0	0.0	0.0	0.0	0.0	0.0	0.0	0.0	0.0	-0.2	0.3	0.0	0.0
AT5G59070	0.6	0.2	0.6	0.1	0.6	0.1	0.6	0.2	0.0	0.4	-0.3	0.2	-0.7	0.1
AT5G59590	0.5	0.1	0.3	0.1	0.1	0.1	0.2	0.1	-0.4	0.4	-0.7	0.1	0.1	0.1
AT5G65310	0.0	0.1	0.0	0.1	0.1	0.1	0.2	0.1	0.1	0.1	0.0	0.1	-0.2	0.0

19_4	Ratio compared with 0d												
Inserted loci	1d/0d	1.5d/0d	2d/0d	2.5d/0d	3d/0d	3.5d/0d	4d/0d	4.5d/0d	5d/0d	7d/0d	9d/0d	11d/0d	13d/0d
AT1G13920	0.0	0.0	0.0	0.0	0.0	0.1	0.1	0.1	0.0	0.1	0.0	0.0	-0.1
AT1G29715	-0.4	-0.2	-0.1	-0.2	-0.3	-0.3	-0.2	-0.4	-0.3	0.0	0.3	0.4	0.3
AT1G66400	0.1	0.2	0.0	0.0	0.1	-0.1	-0.1	-0.2	-0.2	-0.2	-1.1	-0.4	-0.7
AT1G70940	0.0	0.1	0.0	-0.1	-0.2	-0.2	-0.3	-0.4	-0.5	-0.5	-0.4	-0.6	-1.1
AT1G73870	0.0	0.1	0.1	0.0	0.0	0.0	0.1	-0.3	-0.3	-0.8	-1.4	-1.6	-1.6
AT1G76230	-0.1	-0.1	0.0	-0.2	0.1	0.1	0.0	0.0	0.0	-0.1	-0.4	-0.2	-0.2
AT2G35810	0.0	0.0	0.0	0.0	0.0	0.0	0.0	0.0	0.0	0.0	0.0	0.0	-0.1
AT2G40220	-0.1	0.0	-0.3	0.1	0.0	0.0	0.0	-0.5	0.0	0.1	0.1	0.1	-0.1
AT3G47150	0.0	-0.1	0.0	0.0	-0.2	0.0	0.0	0.0	0.0	0.0	0.0	0.0	0.1
AT4G16550	-0.1	-0.3	-0.6	-0.8	-0.9	-1.0	-1.1	-1.1	-1.2	-1.2	-0.5	-0.2	0.1
AT4G24430	-0.1	-0.2	-0.2	-0.2	-0.3	-0.2	-0.3	-0.4	-0.3	-0.7	-1.0	-1.9	-1.0
AT4G25330	0.0	0.1	0.2	0.3	0.3	0.2	0.0	-0.2	-0.4	-0.2	0.7	0.8	0.8
AT5G63710	0.1	0.1	0.2	0.1	0.1	0.3	0.3	0.1	0.0	-0.1	-0.4	-0.7	-0.9

19_4	0d		1d		1.5d		2d		2.5d		3d		3.5d	
Inserted loci	average	s.d	average	s.d	average	s.d	average	s.d	average	s.d	average	s.d	average	s.d
AT1G13920	-0.1	0.1	-0.1	0.1	-0.1	0.0	-0.1	0.1	-0.1	0.0	-0.1	0.0	0.0	0.0
AT1G29715	0.0	0.3	-0.4	0.0	-0.2	0.1	-0.1	0.1	-0.2	0.1	-0.3	0.0	-0.2	0.1
AT1G66400	0.3	0.1	0.3	0.0	0.4	0.2	0.2	0.1	0.3	0.0	0.3	0.1	0.2	0.0
AT1G70940	0.4	0.1	0.4	0.1	0.5	0.1	0.4	0.1	0.3	0.1	0.2	0.1	0.2	0.0
AT1G73870	0.9	0.1	0.9	0.1	0.9	0.2	0.9	0.2	0.9	0.1	0.9	0.1	0.9	0.2
AT1G76230	0.0	0.1	-0.2	0.1	-0.1	0.2	-0.1	0.1	-0.2	0.6	0.0	0.1	0.0	0.0
AT2G35810	0.0	0.1	0.0	0.0	0.0	0.0	0.0	0.1	0.0	0.0	0.0	0.0	0.0	0.1
AT2G40220	-0.1	0.2	-0.2	0.4	-0.1	0.2	-0.4	0.5	0.0	0.0	-0.1	0.2	-0.1	0.2
AT3G47150	0.0	0.0	0.0	0.0	-0.1	0.1	0.0	0.0	0.0	0.0	-0.2	0.3	0.0	0.0
AT4G16550	0.8	0.1	0.7	0.1	0.5	0.1	0.2	0.1	0.0	0.1	-0.1	0.0	-0.2	0.1
AT4G24430	1.0	0.4	0.9	0.1	0.8	0.1	0.8	0.0	0.8	0.1	0.8	0.1	0.8	0.2
AT4G25330	-0.2	0.0	-0.2	0.1	-0.1	0.1	0.0	0.1	0.1	0.1	0.1	0.0	0.0	0.0
AT5G63710	0.3	0.0	0.4	0.1	0.4	0.2	0.5	0.1	0.4	0.1	0.4	0.1	0.6	0.1

19_4	4d		4.5d		5d		7d		9d		11d		13d	
Inserted loci	average	s.d	average	s.d	average	s.d	average	s.d	average	s.d	average	s.d	average	s.d
AT1G13920	0.0	0.0	0.0	0.1	-0.1	0.1	0.0	0.0	-0.1	0.1	-0.1	0.1	-0.2	0.1
AT1G29715	-0.1	0.1	-0.3	0.0	-0.2	0.2	0.0	0.1	0.3	0.3	0.5	0.0	0.4	0.3
AT1G66400	0.1	0.1	0.0	0.1	0.1	0.0	0.0	0.0	-0.9	0.2	-0.2	0.1	-0.4	0.0
AT1G70940	0.1	0.0	0.0	0.0	-0.1	0.0	-0.1	0.1	0.0	0.1	-0.2	0.0	-0.7	0.1
AT1G73870	0.9	0.0	0.6	0.1	0.6	0.1	0.1	0.1	-0.5	0.1	-0.7	0.2	-0.7	0.1
AT1G76230	0.0	0.0	-0.1	0.1	-0.1	0.2	-0.2	0.1	-0.5	0.1	-0.2	0.1	-0.2	0.1
AT2G35810	0.0	0.1	0.0	0.0	0.0	0.1	0.0	0.0	0.0	0.1	0.0	0.0	-0.1	0.0
AT2G40220	-0.1	0.2	-0.6	0.5	-0.1	0.3	0.0	0.1	0.0	0.0	0.0	0.1	-0.2	0.1
AT3G47150	0.0	0.0	0.0	0.0	0.0	0.0	0.0	0.0	0.0	0.0	0.0	0.0	0.1	0.1
AT4G16550	-0.3	0.1	-0.3	0.0	-0.4	0.0	-0.4	0.0	0.3	0.1	0.6	0.0	0.9	0.0
AT4G24430	0.7	0.1	0.6	0.1	0.7	0.1	0.3	0.0	0.0	0.0	-0.9	0.1	0.0	0.1
AT4G25330	-0.2	0.0	-0.4	0.0	-0.6	0.0	-0.4	0.1	0.5	0.2	0.6	0.1	0.6	0.0
AT5G63710	0.6	0.1	0.4	0.1	0.3	0.0	0.2	0.1	-0.1	0.2	-0.4	0.0	-0.6	0.1

Log2 gene expression of Onsen inserted loci was obtained from public data set of heat stress (Ryan et al., 2015, BMC Genomics 16:488)

Log2 normarization was calculated with RMA method , using affy package on R program

Statistic analysis was used with t-test and Benjamini-Hochberg FDR

Red boxes indicate upregulate genes (FDR alpha < 0.05, Log 2 expression ration >1)

Blue boxes indicate down-regulated genes (FDR alpha < 0.05, Log2 expression ratio <-1)

Supplementary Table S6. Primer sequences.

Target/Purpose	Name	Primer
Southern probe	ONSEN-F	TAA TGT TCC CTT CCA AGT CCC
	ONSEN-R	GCT TGT AAT GAC CCA AGA AGT
ABI5 insertion PCR	AT2G36270-F	CAC ACT CTC ACG GTG AGA ACA
	AT2G36270-R	TAC TTC TCG CTC TGA CGT CAA
ABI4 insertion PCR	ABI4 down-F	ACC TCC TCT TCC TCC ACT CAA
	ABI4-R	CCT CTG AAA CTC GAA CAA CCA
5' bisulfite PCR	ABI BS F1	GAT YTT GGA TTA GAA GAY TGT TAA ATA AAT
	COPIA78 BS R1	AAA CCT TTC TCR ACT ATC TCC CAC ACR TCA T
3' bisulfite PCR	COPIA78 BS F1	TTT ATY AAG ATG AGG AGT TTG YTT GGA GTA G
	ABI BS R1	AAA ATA TCT TAA CCC ACT CRT TTT CTT CTT
ABI4 q-RT-PCR	ABI4-qPCR_F	GGG ACA ATT CCA ACA CCA ACA G
	ABI4-qPCR_R	GAA CAT ACC GGA TCA ACC AAC G
ABI5 q-RT-PCR	ABI5qPCR-LP	AAA TTG CCG AAA TCG AAC AG
	ABI5qPCR-RP	TTT TCC CTC GTT CTG TCT CC
ABI5 q-RT-PCR on the *ONSEN* insertion	ABI5_qRTPCR_2-F	ACA CAA AGC CAC CGG TTT TTA
	ABI5_qRTPCR_2-R	AAC GGA TGA TTC TCA CCA CCA
ABI5 1st intron OneStep RT-PCR	ABI5 cDNA-F	TTC AGT CAA AAT TCT CCG GC
	ABI5 cDNA-R	TAA AGA CAC CAA CAC CAC CCT
3’RACE RT	oligodT T7 2-3	CAG TGA ATT GTA ATA CGA CTC ACT ATA GGN VTT TTT TTT TTT TTT T
3’RACE 1st primer	T7-3 primer	TTC CTC ATC AAT GTC CGA A
3’RACE 2nd primer	T7-2 primer	TTG TAA TAC GAC TCA CTA TAG G
Gene specific 1st primer	ABI5 1st	CGG CTT TTA AAC TAT GTG AAG G
Gene specific 2nd primer	ABI5 2nd	CCA TAA CAA GAA GCG GAT TCT C
ONSEN copy in *ABI4*	AT2G40220-F	CAA CAT CTT CCT TAA TGG TGG GA
	CopiaMix-R	TGA TCT CAA TTC TTC AAT CG
ONSEN copy in *ABI5*	AT2G36270seq-part2F	TGA AGG AGG AGA ACC TCC ATA
	CopiaMix-R	TGA TCT CAA TTC TTC AAT CG
